# Protein-Trap Insertional Mutagenesis Uncovers New Genes Involved in Zebrafish Skin Development, Including a *Neuregulin 2a*-Based ErbB Signaling Pathway Required during Median Fin Fold Morphogenesis

**DOI:** 10.1371/journal.pone.0130688

**Published:** 2015-06-25

**Authors:** Stephanie E. Westcot, Julia Hatzold, Mark D. Urban, Stefânia K. Richetti, Kimberly J. Skuster, Rhianna M. Harm, Roberto Lopez Cervera, Noriko Umemoto, Melissa S. McNulty, Karl J. Clark, Matthias Hammerschmidt, Stephen C. Ekker

**Affiliations:** 1 Department of Genetics, Cell Biology, and Development, University of Minnesota, Minneapolis, Minnesota, United States of America; 2 Department of Biochemistry and Molecular Biology, Mayo Clinic, Rochester, Minnesota, United States of America; 3 Institute for Developmental Biology, University of Cologne, Biocenter, Cologne, Germany; 4 Center for Molecular Medicine Cologne (CMMC), University of Cologne, Cologne, Germany; 5 Cologne Cluster of Excellence in Cellular Stress Responses in Aging-associated Diseases (CECAD), University of Cologne, Cologne, Germany; Institute of Molecular and Cell Biology, SINGAPORE

## Abstract

Skin disorders are widespread, but available treatments are limited. A more comprehensive understanding of skin development mechanisms will drive identification of new treatment targets and modalities. Here we report the Zebrafish Integument Project (ZIP), an expression-driven platform for identifying new skin genes and phenotypes in the vertebrate model *Danio rerio* (zebrafish). *In vivo* selection for skin-specific expression of gene-break transposon (GBT) mutant lines identified eleven new, revertible GBT alleles of genes involved in skin development. Eight genes—*fras1*, *grip1*, *hmcn1*, *msxc*, *col4a4*, *ahnak*, *capn12*, and *nrg2a*—had been described in an integumentary context to varying degrees, while *arhgef25b*, *fkbp10b*, and *megf6a* emerged as novel skin genes. Embryos homozygous for a GBT insertion within *neuregulin 2a* (*nrg2a*) revealed a novel requirement for a Neuregulin 2a (Nrg2a) – ErbB2/3 – AKT signaling pathway governing the apicobasal organization of a subset of epidermal cells during median fin fold (MFF) morphogenesis. In *nrg2a* mutant larvae, the basal keratinocytes within the apical MFF, known as ridge cells, displayed reduced pAKT levels as well as reduced apical domains and exaggerated basolateral domains. Those defects compromised proper ridge cell elongation into a flattened epithelial morphology, resulting in thickened MFF edges. Pharmacological inhibition verified that Nrg2a signals through the ErbB receptor tyrosine kinase network. Moreover, knockdown of the epithelial polarity regulator and tumor suppressor *lgl2* ameliorated the *nrg2a* mutant phenotype. Identifying Lgl2 as an antagonist of Nrg2a – ErbB signaling revealed a significantly earlier role for Lgl2 during epidermal morphogenesis than has been described to date. Furthermore, our findings demonstrated that successive, coordinated ridge cell shape changes drive apical MFF development, making MFF ridge cells a valuable model for investigating how the coordinated regulation of cell polarity and cell shape changes serves as a crucial mechanism of epithelial morphogenesis.

## Introduction

Skin conditions generate between 12% to 43% of medical visits [1, 2]. In the United States, skin disorders are estimated to affect one third of the population at any time, with an estimated total annual cost of nearly US$100 billion [3]. Patients with skin disease frequently suffer physical discomfort and pain, and often experience diminished quality of life and psychological distress [4–6]. Medically, skin conditions are challenging to treat: skin is an exposed, physically vulnerable external barrier whose continuous turnover can impede long-lasting healing. Because there is a limited variety of clinical treatment methods, many of which are non-specific immune modulators such as steroids [7], new therapeutic targets for skin conditions could have important health and economic benefits [8]. Strategies for identifying novel integument genes and expanding our understanding of incompletely characterized integument loci offer avenues for subsequent interventional approaches.

**Fig 1:**
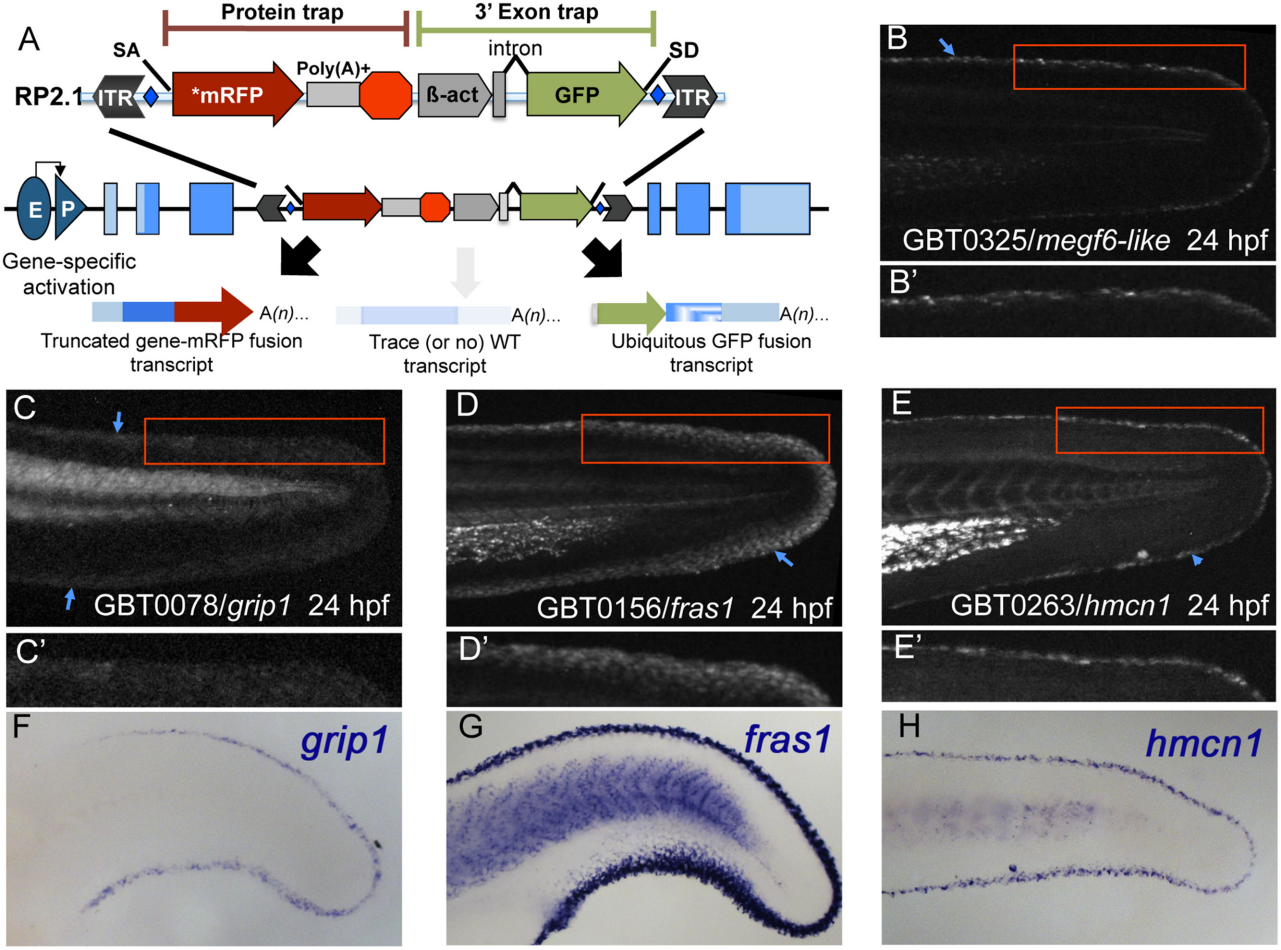
Gene-break transposon—based protein trapping identifies known and new epidermal median fin fold loci. (A) A schematic of the RP2.1 gene-break transposon (GBT) vector used in this study. Gene-breaking activity occurs when an endogenous locus with a GBT insertion is transcribed. The vector-supplied splice acceptor (SA) in the 5’ protein trap cassette intercepts the endogenous splicing machinery and transcript, redirecting them to read directly into an AUG-free mRFP sequence (*mRFP). That event generates a fusion transcript by tagging the 5’ portion of the endogenous transcript with mRFP. When translated, the mRFP fusion transcript will produce a potentially mutagenic truncated protein product. Simultaneously, the 3’ exon trap cassette uses the vector-supplied splice donor (SD) to create a GFP fusion transcript with the remaining downstream endogenous transcript. GBT alleles are revertible because loxP sites (blue diamonds) flank the cassettes, allowing the mutagenic elements to be excised in the presence of Cre recombinase. (B-E’) At 24 hours post-fertilization (24 hpf), GBT-generated mRFP fusion proteins from *megf6a* ^mn0325Gt^ (B), *grip1* ^mn0078Gt^ (C), *fras1* ^mn0156Gt^ (D), and *hmcn1* ^mn0263Gt^ (E) localize along MFF edges. (B’, E’) Both *megf6a* ^mn0325Gt^ (B’) and *hmcn1* ^mn0263Gt^ (E’) localize within a narrow region along the MFF edge (blue arrowheads). (C’, D’) *grip1* ^mn0078Gt^ (C’) and *fras1* ^mn0156Gt^ (D’) localization also follows the fin fold edge (blue arrowheads), though they are distributed somewhat more diffusely than are *megf6a* ^mn0325Gt^ (B’) and *hmcn1* ^mn0263Gt^ (E’). (F-H) Whole-mount *in situ* hybridization (WISH) in 24 hpf wild-type embryos reveals similar MFF expression patterns of endogenous *grip1* (F), *fras1* (G), and *hmcn1* (H) genes. The mRFP fusion protein localization patterns observed in the respective GBT lines recapitulate endogenous gene expression (C, D, E).

The zebrafish (*Danio rerio*) is an excellent vertebrate model for understanding development and disease. Zebrafish not only share significant genomic similarity with humans [9, 10], they also generate numerous transparent, externally fertilized embryos ideal for *in vivo* imaging and for phenotype-based gene discovery (“forward genetics”) [11, 12]. In addition to traditional chemical mutagenesis [13, 14], forward genetic screening uses insertional mutagenesis methods, including retroviruses [15, 16] and the more recently developed gene-breaking transposon (GBT) technology (Fig 1A) [17]. GBT mutagenesis generates mRFP-tagged, Cre recombinase-revertible insertional alleles with ≥ 97% knockdown of endogenous transcript levels [17]. Zebrafish GBT insertional mutagenesis has already identified and characterized new genes, expression patterns, and phenotypes in the heart [18, 19], vasculature [20], and muscle [21]. GBT insertional mutagenesis has also been used to dissect genetic links between brain and behavior [22]. However, it had not previously been applied to studying skin biology.

Zebrafish larval skin and mammalian fetal skin share many features integral to both epidermal biology and disease. In both cases, the developing epidermis is a bilayered epithelium. The outer enveloping layer (EVL) of zebrafish embryos and larvae corresponds to mammalian embryonic periderm [23, 24], while the inner layer consists of basal keratinocytes that will eventually generate the multi-layered mature epidermis [25–27]. In both mammals and zebrafish, the transcription factor DeltaNp63 (ΔNp63) is crucial for epidermal specification and promotes basal keratinocyte proliferation and stemness [28–34].

Fin fold development is an important event in larval epidermal development. Zebrafish larvae have an unpaired median fin fold (MFF) and a pair of pectoral fin folds (PFFs). The MFF is an ancient structure whose origins predate the evolution of paired pectoral fins [35, 36]. MFF development begins at 18 hours post-fertilization (hpf) when midline basal keratinocytes undergo profound cell shape changes that drive median epidermal ridge (MER) formation along the ventral and dorsal caudal midlines. Midline basal keratinocytes adopt a wedge-shaped cross-sectional profile by reducing their basal surfaces and expanding their apical surfaces. Those shape changes, in conjunction with loss of contact with the underlying mesoderm, push up the midline basal keratinocytes (ridge cells) to create the MER [37, 38]. Additional keratinocytes are then recruited to the proximal base of the MER. There they initiate MFF formation by elevating the MER perpendicularly to the larval mediolateral body axis. As the MFF extends further from the larval body, a sub-epidermal (dermal) space forms between the resulting apposed epidermal sheets. During MFF extension, the ridge cells of the initial MER remain at the tip of the MFF. As we show here, ridge cells’ basal surfaces have re-expanded by 30 hpf during a second phase of cell shape changes. The single cells at the tip of the apical MFF (cleft cells) maintain an overall wedge shape while the growing basal surface invaginates, forming an intracellular cleft as a distal extension of the dermal space. In contrast, progressive apical and basal surface growth leads the two to three ridge cells on either side of the cleft cell to elongate, adopting a flattened, epithelial morphology with a rectangular cross-sectional profile.

Little is known about the genetic control of the processes that direct MER formation [38, 39], but studies indicate that MER cells play crucial roles during MFF development and maintenance. As MFF development proceeds, MFF basal keratinocytes, including MER cells, secrete extracellular components into the dermal space. Those components then assemble into specialized extracellular structures, including cross-fibers [37, 40] and collagenous actinotrichia [41, 42], which provide the MFF with structural support against mechanical stresses. While basal keratinocytes along the entire proximo-distal length of the MFF secrete actinotrichia components [42], MER cleft and ridge cells are the predominant producers of basement membrane (BM) components such as Laminin [43, 44] (see below) and BM-dermal anchorage proteins such as Fras1 [44, 45] (see below). By 48 hpf, fin mesenchymal cells (FMCs), a type of dermal fibroblast, have finished invading the MFF to further support and organize the dermal space and its ECM [40, 46].

Genetic evidence increasingly demonstrates that zebrafish MFF development models multiple processes affected in human skin diseases. Two prominent examples are *fraser syndrome 1* (*fras1*) and *fraser-related extracellular matrix 2a* (*frem2a*), encoding extracellular matrix (ECM) components mutated in human Fraser Syndrome patients. During human and mouse development, correct BM-dermal junction formation requires FRAS1 and FREM2, especially in limb buds and craniofacial regions. *FRAS1* and *FREM2* mutations cause transient, locally restricted fetal skin blistering and later lead to characteristic malformations such as cryptopthalmos and syndactyly [47–49]. Likewise, mutations in the corresponding zebrafish homologs *fras1* and *frem2a* lead to transient skin blistering in developing larval fins [44]. Additional examples are the zebrafish mutants in the BM component *laminin α5* (*lama5*) [43] and its transmembrane receptor *integrin α3* (*itga3*) [44], as well as in the intracellular integrin modulator *kindlin-1* (*fermt1*) [50]. All of these mutants are characterized by impaired MFF epidermal integrity, and they model the corresponding epidermal defects characteristic of human epidermolysis bullosa (*lama5*, *itga3*) and Kindler Syndrome (*fermt1*).

Our study used GBT technology to identify eleven zebrafish integument loci expressed in either larval epidermis or FMCs. Three of those loci (*fras1*, *hmcn1*, *grip1*) are novel revertible alleles of known disease-related genes that will provide new, Cre-regulatable molecular tools for skin biology studies. We also identified *neuregulin 2a* (*nrg2a*) as a novel essential regulator of apical MFF development and MFF ridge cell polarity. *nrg2a* is a zebrafish homolog of the epidermal growth factor (EGF) superfamily member *NEUREGULIN 2* (*NRG2*). Although an earlier morpholino (MO) knockdown study indicates that *nrg2a* participates in dorsal root ganglion development [51], *nrg2a* has not been investigated in conjunction with skin or MFF development. We show here that zebrafish *nrg2a* ^mn0237Gt/mn0237Gt^ insertional mutants display specific defects in MFF ridge cell organization, leading to aberrant apical MFF morphology reminiscent of ErbB2 (*erbb2* ^-/-^) mutants [52]. ErbB receptor tyrosine kinases (RTKs) mediate signaling by EGF superfamily ligands, such as the Neuregulins (NRGs), through several different signal transduction pathways, including the mitogen-activated protein kinase (MAPK) ERK, and/or phosphatidylinositol-3-kinase (PI3K) and AKT [53]. Our pharmacological inhibition, *erbb* expression, and activated ERK (pERK) and AKT (pAKT) immunofluorescence studies were consistent with this novel Nrg2a-dependent MFF pathway operating through ErbB2/3 and the PI3K –AKT signal transduction cascade. Furthermore, the *nrg2a* phenotype led us to discover an early role for the epithelial cell polarity regulator and tumor suppressor *lethal giant larvae 2* (*lgl2*) in MFF development. Previous mutant studies established that *lgl2* promotes epidermal integrity by attenuating ErbB signaling during late larval development (96–120 hpf), but did not find an earlier developmental role for *lgl2* despite having documented its expression from 24 hpf onwards [52, 54]. We show here that MO-directed knockdown of *lgl2* in *nrg2a*-deficient larvae rescues the MFF defects caused by loss of functional Nrg2a two days before *lgl2*’s tumor suppressor role. Therefore, we have identified a previously undocumented negative impact of *lgl2* on ErbB signaling that regulates epithelial polarity and keratinocyte biology during epidermal morphogenesis in the apical fin fold.

## Results

### Gene-breaking Transposon (GBT) alleles of eleven genes with previously known and unknown expression in epidermal or fin mesenchymal cells

**Fig 2:**
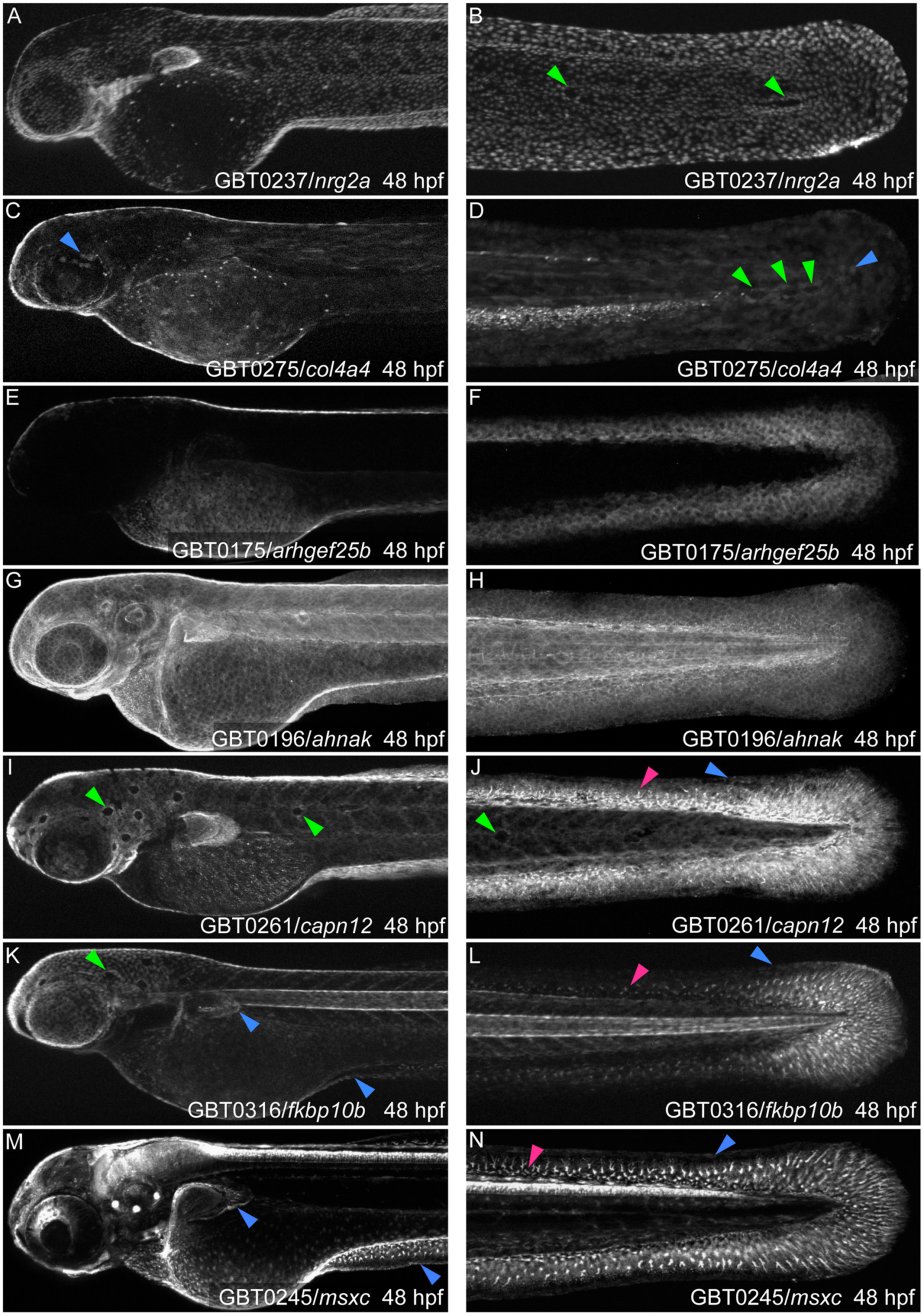
GBT protein trapping identifies integument loci with epidermal or fin mesenchymal expression. As identified by mRFP localization, seven GBT alleles have unique epidermal expression patterns emphasizing epidermal continuity over the body and larval fin folds. They are: (A, B) *nrg2a* ^mn0237Gt^, (C, D) *col4a4* ^mn0275Gt^, (E, F) *arhgef25b* ^mn0175Gt^, (G, H) *ahnak* ^mn0196Gt^, (I, J) *capn12* ^mn0261Gt^, (K, L) *fkbp10b* ^mn0316Gt^, and (M, N) *msxc* ^mn0245Gt^. (B, D, I, J, K) Four lines, *nrg2a* ^mn0237Gt^, *col4a4* ^mn0275Gt^, *capn12* ^mn0261Gt^, and *fkbp10b* ^mn0316Gt^, have “holes” or “gaps” in their epidermal pattern (green arrowheads) where neuromasts embedded in the basal layer exclude the mRFP-positive basal keratinocytes. (A, G, I, K, M) *nrg2a* ^mn0237Gt^ (A), *ahnak* ^mn0196Gt^ (G), *capn12* ^mn0261Gt^ (I), *fkbp10b* ^mn0316Gt^ (K), and *msxc* ^mn0245Gt^ (M) are also expressed in the pectoral fin folds. (C-D, J, L, M-N) *col4a4* ^mn0275Gt^, *capn12* ^mn0261Gt^, *fkbp10b* ^mn0316Gt^, and *msxc* ^mn0245Gt^ epidermal expression (blue arrowheads) can be difficult to discern among other expression pattern components. (I-N) *capn12* ^mn0261Gt^, *megf6a* ^mn0316Gt^, and *msxc* ^mn0245Gt^ also show expression in fin mesenchymal cells (pink arrowheads). (C, D, K, L, M, N) Several lines are also expressed in other tissues. (C, D) *col4a4* ^mn0275Gt^ appears in myotomes and the vascular plexus. (K, L) *fkbp10b* ^mn0316Gt^ is seen in the notochord. (M, N) *msxc* ^mn0245Gt^ is strongly expressed in the brain, spinal cord, and sensory maculae. hpf, hours post-fertilization. Comparisons of the mRFP localization patterns with the expression pattern of the endogenous genes are shown in Supporting Information (S1 Fig)

**Fig 3:**
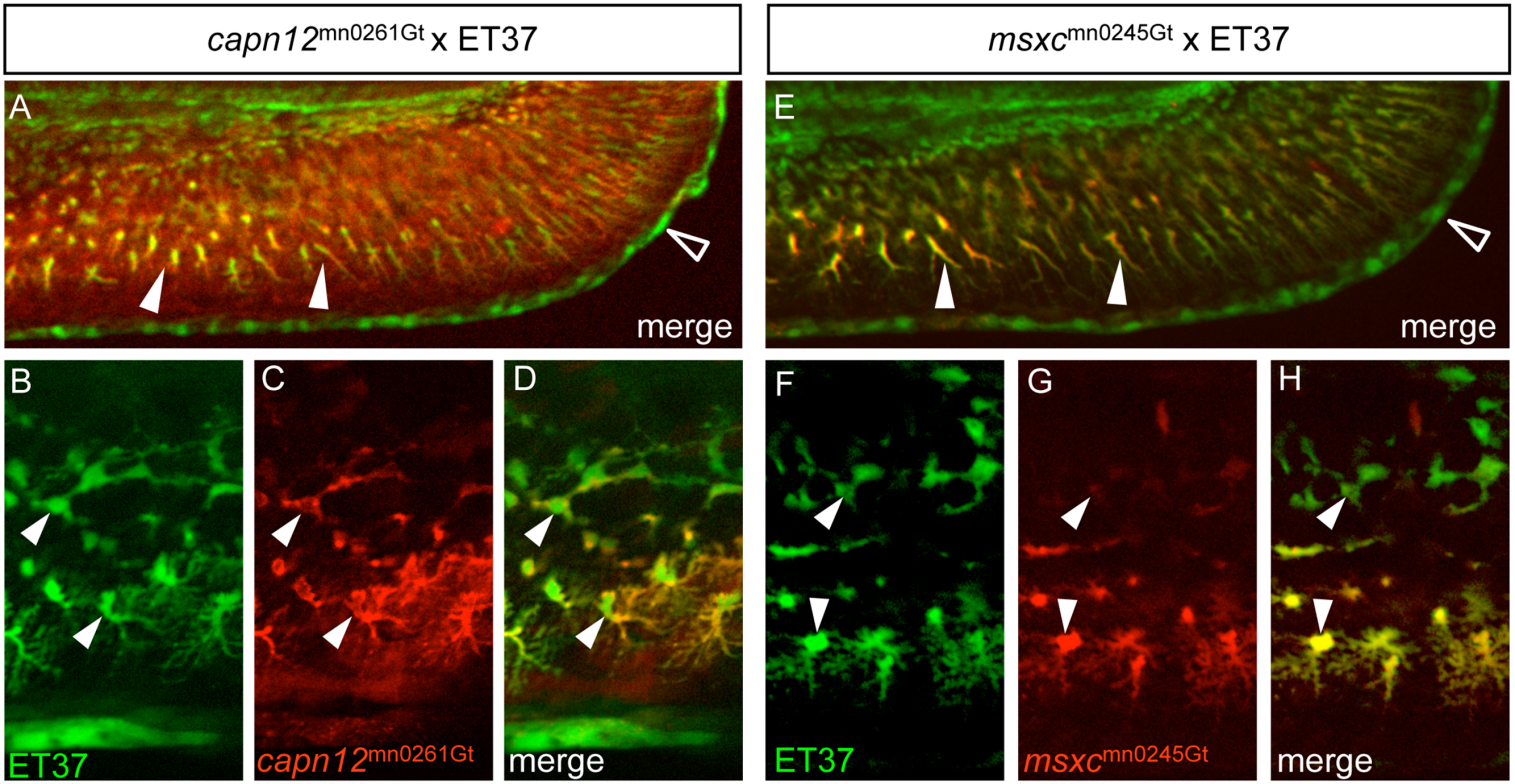
*capn12* and *msxc* transgenes are co-expressed with a fin mesenchymal cell marker. (A, E) When *capn12* ^mn0261Gt^ and *msxc* ^mn0245Gt^ are crossed into an ET37 background, both Capn12^mn0261Gt^-mRFP and MsxC^mn0245Gt^-mRFP co-localize with ET37 EGFP-positive fin mesenchymal cells (FMCs) (arrowheads). ET37 also labels the cleft cells along the MFF edge (open arrowheads). Neither Capn12^mn0261Gt^-mRFP nor MsxC^mn0245Gt^-mRFP localizes to the cleft cells. (B-H) ET37 EGFP labels all FMCs within the fin (arrowheads), both proximal (upper arrowheads) and distal (lower arrowheads), and is present throughout FMC cell bodies and in the extended “arbors” of distal FMCs (lower arrowheads). Both Capn12^mn0261Gt^-mRFP and MsxC^mn0245Gt^-mRFP localization show similarities with and differences from ET37 EGFP. (B-D) Both ET37 EGFP and Capn12^mn0261Gt^-mRFP are expressed in proximal and distal FMCs (arrowheads) but unlike ET37 EGFP, Capn12^mn0261Gt^-mRFP localizes to the periphery of FMCs (arrowheads). (F-H) MsxC^mn0245Gt^-mRFP co-localizes with ET37 EGFP, but differs from Capn12^mn0261Gt^-mRFP. MsxC^mn0245Gt^-mRFP is much weaker in proximal FMCs (upper arrowheads) than in distal FMCs (lower arrowheads). Unlike Capn12^mn0261Gt^-mRFP, MsxC^mn0245Gt^-mRFP subcellular localization is not noticeably distinct from that of ET37 EGFP.

Locating expression within a tissue of interest is a key step toward identifying new genes involved in tissue-specific biology. Using the online GBT database and image catalog zfishbook.org (http://www.zfishbook.org) [55] as an initial selection tool, we screened 350 GBT insertional alleles [17] for mRFP expression within the skin of the MFF (Fig 1B–1E; Fig 2B, 2D, 2F, 2H, 2J, 2L, and 2N) and/or covering the larval body (Fig 2A, 2C, 2E, 2G, 2I and 2K) [37, 38, 56]. That expression prioritization screen identified eleven gene-break alleles (GBT lines) with mRFP localization in the integument (Fig 1B–1E; Fig 2). We refer to these as the initial Zebrafish Integument Project (ZIP) GBT lines. The ZIP lines’ RFP localization patterns [17] fell into three broad categories. The first was expression in MFF edges (Fig 1B–1E); the second was expression throughout some or all of the epidermis (Fig 2A–2N); and the third was expression in FMCs (Fig 2I–2N; Fig 3). Because we identified these eleven ZIP lines among the initial cohort of GBT insertional alleles, the GBT-disrupted genes in several ZIP lines had been identified during our initial GBT mutagenesis study [17]. We completed sequence-based identification of ZIP genes not identified in the previous study (Table 1).

**Table 1:** Gene identification.

Line ID	Allele	Gene Name	Locus	LG	Homolog	Type	Position (GRCz10)	Mutant
GBT0078^**§**^	mn0078Gt	*glutamate receptor interacting protein 1*	*grip1*	4	GRIP1^d^	5’ RACE tag	Chr4:13,394,068.. 13,139,114	
GBT0156^**§**^	mn0156Gt	*fraser syndrome 1*	*fras1* *	5	FRAS1^d^	INV/ TAIL	Chr5:38,361,783.. 38,362,256	*pinfin* (*pif*)
GBT0175^**§**^ ^**‡**^	mn0175Gt	*rho guanine nucleotide exchange factor (GEF) 25b*	*arhgef25b*	6	ARHGEF25	INV/ TAIL	Chr6:39,272,998.. 39,272,990	
GBT0196	mn0196Gt	*ahnak*	*ahnak*	14	AHNAK	INV/ TAIL	Chr14:26,478,533.. 26,478,540	
GBT0237	mn0237Gt	*neuregulin 2a*	*nrg2a*	21	NRG2	INV/ TAIL	Chr21:28,628,953.. 28,628,961	*neuregulin 2a (nrg2a*)
GBT0245	mn0245Gt	*muscle segment homeobox C*	*msxc*	13	Msx3	NIH	Chr13:24,532,473.. 24,532,481	
GBT0261	mn0261Gt	*calpain 12*	*capn12*	18	CAPN12	5' RACE tag	Chr18:36,714,060.. 36,731,572	
GBT0263	mn0263Gt	*hemicentin 1*	*hmcn1* *	20	HMCN1	NIH	Chr20:34,304,041.. 34,304,049	*nagel* (*nel*)
GBT0275	mn0275Gt	*collagen 4a4*	*col4a4*	15	COL4A4	NIH	Chr15:36,095,393.. 36,095,386	
GBT0316^**‡**^	mn0316Gt	*FK506 binding protein 10b*	*fkbp10b*	12	FKBP10	INV/ TAIL	Chr12:13,870,717.. 13,870,724	
GBT0325^**§**^ ^**‡**^	mn0325Gt	*multiple EGF-like domains protein 6a*	*megf6a*	11	MEGF6	NIH	Chr11:41,137,656.. 41,137,664	

We identified the corresponding GBT-tagged locus for each ZIP line. Consistent with the random nature of RP2 GBT insertion, no two insertions were on the same linkage group (LG). All eleven ZIP loci have mammalian homologs. Ten have human homologs, and one (*msxc* ^mn0245Gt^) has a murine homolog but no reported human homolog. Mutations in two of those ten human homologs, *FRAS1* and *GRIP1*, are known to be involved in Fraser Syndrome, a human disease whose phenotype includes blistering. Three ZIP alleles are novel integument genes: *arhgef25b* ^mn0175Gt^, *fkbp10b* ^mn0316Gt^, and *megf6a* ^mn0325Gt^. Although several ZIP alleles (§) were reported in an earlier publication, this study initially identified their expression patterns and includes previously unpublished data for each.

^§^ Sequence identified in Clark *et al*., 2011 [17].

^‡^ Novel integument genes.

* GBT homozygous mutant phenocopies published ENU mutant at the designated locus.

^d^ Human homolog is a disease-related gene.

Four ZIP lines displayed restricted mRFP localization in epidermal cells along MFF edges at 24 hpf: GBT0078/*glutamate receptor interacting protein 1* (*grip1* ^mn0078Gt^; Fig 1C and 1C’), GBT0156/*fraser syndrome 1* (*fras1* ^mn0156Gt^; Fig 1D and 1D’), GBT0263/*hemicentin 1* (*hmcn1* ^mn0263Gt^; Fig 1E and 1E’), and GBT0325/*multiple epidermal growth factor-like domains 6a* (*megf6a* ^mn0325Gt^; Fig 1B and 1B’). As we had expected, the *grip1* ^mn0078Gt^, *fras1* ^mn0156Gt^, and *hmcn1* ^mn0263Gt^ alleles’ respective mRFP localization patterns, as well as their respective *mRFP* fusion transcript expression data from whole-mount *in situ* hybridization (WISH) [57, 58] (Fig 1F–1H), corresponded to previous *in situ* transcript expression data [17, 44, 45]. Thus our data affirmed that these gene-break alleles reliably recapitulated endogenous expression of the trapped loci.

Seven lines displayed global epidermal localization throughout the entire integument: GBT0237/*neuregulin 2a* (*nrg2a* ^mn0237Gt^; Fig 2A and 2B), GBT0275/*collagen 4a4* (*col4a4* ^mn0275Gt^; Fig 2C and 2D), GBT0175/*arhgef25b* (*arhgef25b* ^mn0175Gt^; Fig 2E and 2F), GBT0196/*ahnak* (*ahnak* ^mn0196Gt^; Fig 2G and 2H), GBT0261/*calpain 12* (*capn12* ^mn0261Gt^; Fig 2I and 2J), GBT0316/*fk 506 binding protein 10b* (*fkbp10b* ^mn0316Gt^; Fig 2K and 2L), and GBT0245/*muscle segment homeobox C* (*msxc* ^mn0245Gt^; Fig 2M and 2N). Several expression patterns emphasized external topology, especially where the larval epidermis covered the head and eye (Fig 2A, 2C, 2I, 2J and 2K), the yolk and yolk extension (Fig 2A, 2C, 2E, 2G, 2I, 2K and 2M), the pectoral fin folds (Fig 2A, 2E, 2G, 2I, 2K and 2M), and the median fin fold (Fig 2B, 2D, 2F, 2H and 2L). In contrast, mRFP expression was excluded from neuromasts embedded within the basal layer [59] (*nrg2a* ^mn0237Gt^, Fig 2B; *col4a4* ^mn0275Gt^, Fig 2D; *capn12* ^mn0261Gt^, Fig 2I and 2J; and *fkbp10b* ^mn0316Gt^, Fig 2K).

We observed irregularly shaped mRFP^+^ cells within the fin folds of lines *msxc* ^mn0245Gt^ (Fig 2M and 2N), *capn12* ^mn0261Gt^ (Fig 2I and 2J), and *fkbp10b* ^mn0316Gt^ (Fig 2K and 2L), and the endogenous transcript patterns revealed via WISH analyses supported those observations (S1D’ and S1F’ Fig). Hypothesizing that those mRFP^+^ cells were FMCs [40], we crossed *capn12* ^mn0261Gt^ and *msxc* ^mn0245Gt^ into ET37, a transgenic marker line expressing EGFP in FMCs (among other cell types, such as cleft cells) [40, 60]. Both Capn12^mn0261Gt^-mRFP and Msxc^mn0245Gt^-mRFP fusion proteins co-localized with ET37 EGFP expression in FMCs (Fig 3A, 3D, 3E and 3H). Capn12^mn0261Gt^-mRFP was expressed in FMCs throughout the fin fold (Fig 3C and 3D), but localized to the periphery of FMC cell bodies (Fig 3C and 3D), whereas ET37 EGFP appeared uniformly distributed (Fig 3F and 3H). In contrast, Msxc^mn0245Gt^-mRFP was predominately expressed in FMCs within the distal fin fold (Fig 3B–3D) and was uniformly distributed within individual cells (Fig 3B–3D). Those results confirmed *msxc* ^mn0245Gt^ mesenchymal expression and established *capn12* ^mn0261Gt^ as a new FMC marker for future studies of that migratory cell population.

Several alleles also displayed non-epidermal mRFP localization. Col4a4^mn0275Gt^-mRFP was expressed in the caudal vascular plexus (Fig 2D) and in some somitic myotomes (Fig 2C and 2D). *fkbp10b* ^mn0316Gt^ was expressed in the notochord (Fig 2K and 2L; S1D’ Fig). And consistent with previous reports [61] and with our own WISH analyses of the endogenous *msxc* transcript (S1F Fig), Msxc^mn0245Gt^-mRFP was expressed in the central nervous system (CNS), the sensory maculae of the inner ears, and the pectoral fin buds (Fig 2M and 2N).

Of the eleven genes identified (Table 1; Fig 1B–1D’; Fig 2), three have established roles in skin development and epidermal-dermal junction formation: *fras1*, *grip1*, and *hmcn1* [44, 62]. As described above, FRAS1 is a large basement membrane (BM)-associated ECM protein connecting BM with underlying dermal connective tissue. The cytoplasmic protein GRIP1 is required for properly localizing FRAS1 to the basal side of epithelial cells [63]. In human and mouse, recessive mutations in *FRAS1* [47, 48] and *GRIP1* [63–65] are characterized by malformations resulting from epidermal/epithelial blistering during fetal development. Zebrafish *fras1* mutants are characterized by corresponding blistering in the developing fin folds, though zebrafish *grip1* mutants have no overt morphological phenotype [44]. Hmcn1/Fibulin 6 is a highly conserved member of the Fibulin ECM protein family, with epithelial cell-anchoring roles in *C*. *elegans* [66]. Zebrafish genetic studies have shown Hmcn1 is essential for epithelial-dermal anchorage in developing fin folds [44].

To varying degrees, five other ZIP alleles have reported zebrafish or mammalian integument expression: *col4a4*, *ahnak*, *capn12*, *nrg2a*, and *msxc*. Col4a4 encodes one of the six subunits of type IV collagen, a major BM structural component [8, 62, 67]. Originally isolated from bovine muzzle epidermis as Desmoyokin, AHNAK [68] shuttles between the cytoplasm and the plasma membrane in keratinocytes and other epithelial cells [69]. Ahnak^mn0196Gt^–mRFP shows similar membrane localization (Fig 2G and 2H). CALPAIN12, a member of the Calpain family of calcium-dependent, non-lysosomal cysteine proteases, is expressed in murine skin [70]. Calpain activity has also been implicated in wound healing [71]. Transcriptional profiling data indicate that *NEUREGULIN 2* (*NRG2*) is expressed in the epidermal basal layer [72]. *msxc* encodes the transcription factor MsxC and is expressed in zebrafish FMCs [39, 61].

Finally, ZIP lines *arhgef25b*, *fkbp10b*, and *megf6a* did not have documented integument expression. *arhgef25b* ^mn0175Gt^, *fkbp10b* ^mn0316Gt^, and *megf6a* ^mn0325Gt^ therefore represent novel skin genes—and are novel revertible alleles suitable for future genetic studies.

### *fras1* and *hmcn1* GBT alleles phenocopy known ENU mutants and are revertible

**Fig 4:**
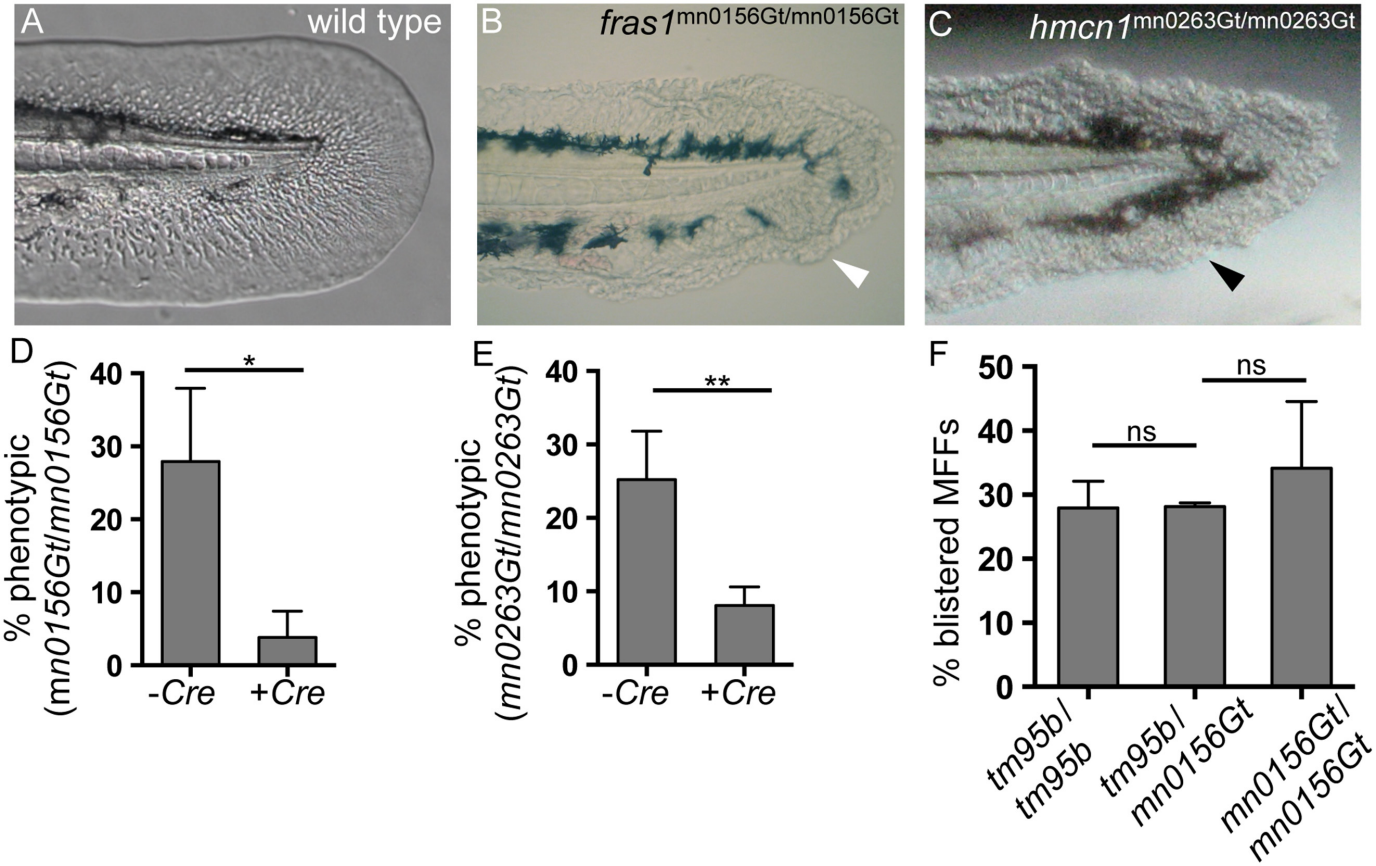
GBT protein trapping generates novel revertible alleles of known MFF loci. (A) By 48 hpf, wild-type MFFs are thin and flat, and the MFF edge appears smooth and regular. (B-C) Two ZIP gene-break alleles, *fras1* ^mn0156Gt^ and *hmcn1* ^mn0263Gt^ have homozygous recessive phenotypes, each of which results in blistered MFFs (arrowheads). (D-E) Both *fras1* ^mn0156Gt^ and *hmcn1* ^mn0263Gt^ behaved as classic revertible GBT mutant alleles in Cre reversion experiments. With both alleles, significantly fewer *Cre* mRNA-injected embryos were phenotypic, compared with their respective uninjected siblings. (D) For offspring of *fras1* ^mn0156Gt^ heterozygote incrosses, 27% (n = 156) of uninjected embryos (-*Cre*) were phenotypic; only 5% (n = 140) of *Cre* mRNA-injected siblings (+*Cre*) were phenotypic (p < 0.05). (E) For offspring of *hmcn1* ^mn0263Gt^ heterozygote incrosses, 25% (n = 146) of uninjected embryos (-Cre) were phenotypic; only 8% (n = 233) of Cre-injected siblings (+Cre) were phenotypic (p < 0.005). (F) The *fras1* ^mn0156Gt^ GBT allele fails to complement the *pif* ^tm95b^ ENU allele of *fras1*. Crossing *pif* ^tm95b^ heterozygotes with *fras1* ^mn0156Gt^ heterozygotes does not reduce the proportion of phenotypic offspring (28%, n = 445) (*tm95b/mn0156Gt* trans-heterozygotes) compared to either *pif* ^tm95b/tm95b^ (*tm95b*/*tm95b*, n = 332) or *fras1* ^mn0156Gt/mn0156Gt^ (*mn0156Gt*/*mn0156Gt*, n = 206) homozygotes. Percentages represent the mean of means (MOM); error bars represent standard deviations (SD).

**Fig 5:**
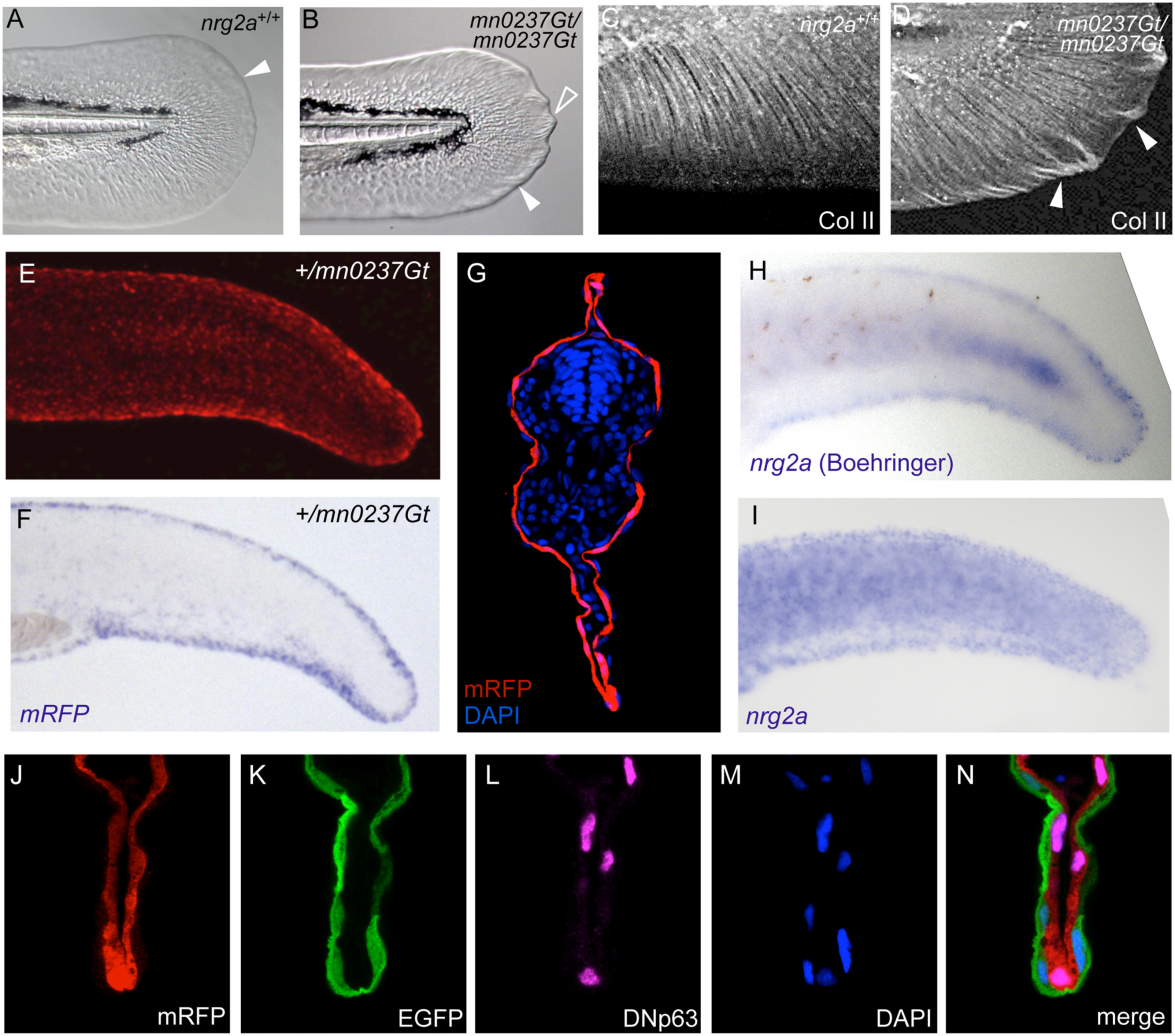
*nrg2a* mutants display altered MFF morphology, consistent with the epidermal localization of the Nrg2-mRFP fusion protein and of endogenous *nrg2a* transcripts. (A, B) By 48 hpf, *nrg2a* ^mn0237Gt/mn0237Gt^ mutants (*mn0237Gt*/*mn0237Gt*) show altered MFF morphology. (A) Wild-type MFF edges are thin, flat, and continuously curved (arrowhead). (B) *mn0237Gt*/*mn0237Gt* mutant MFFs have thickened edges (arrowhead), and one or more pointed protrusions (open arrowhead). (C, D) Collagen II (Col II) immunostaining of actinotrichia support fibers within the MFF shows aberrant collagen accumulation and ectopic actinotrichia within *mn0237Gt*/*mn0237Gt* mutant apical MFFs (arrowheads) at 48 hpf. (E, G) At 24 hpf, Nrg2a-mRFP fusion protein is present in MFFs of heterozygous (+/*mn0237Gt*) embryos (E; view on tail of whole mount) and, at slightly lower levels, throughout the entire epidermis (G; section through tail region; immunostained for RFP and counterstained with DAPI). (F, H, I) Whole-mount *in situ* hybridization (WISH) demonstrates strong MFF expression of the GBT-generated fusion transcript (*mRFP*; F) in a representative +/*mn0237Gt* embryo at 24 hpf. When developed with Boehringer Blocking Reagent, WISH staining for endogenous *nrg2a* transcripts in 24 hpf wild-type embryos also revealed strong MFF expression of the endogenous gene (H, Boeringer). When developed without Boehringer Blocking Reagent, WISH staining further reveals uniform expression of the endogenous *nrg2a* gene throughout the entire epidermis (I). For cross-sections, see Honjo *et al*. (2008), Fig 6C [51]). (J-N) Co-labeling of a transverse section through the dorsal MFF of a +/*mn0237Gt* embryo at 24 hpf reveals restricted localization of the Nrg2a-mRFP fusion protein (J) in ΔNp63-positive basal keratinocytes (L), whereas the outer enveloping layer, labeled with EGFP (K), lacks the Nrg2a-mRFP protein; (M) DAPI counterstain; (N) merged image of different channels shown in (J-M).

To determine which ZIP loci were required for early zebrafish skin or fin fold development, we bred each expressing insertion to homozygosity through a standard inbreeding scheme. For each line, we screened offspring of intercrossed heterozygous parents during the first five days of development (120 hpf) for abnormal skin or fin fold morphology present only in mRFP-positive (GBT-expressing) larvae and absent from their wild-type siblings [17] (an “mRFP-linked” phenotype). Of the eleven lines screened, three displayed recessive, mRFP-linked phenotypes: *fras1* ^mn0156Gt^ (Fig 4B), *hmcn1* ^mn0263Gt^ (Fig 4C), and *nrg2a* ^mn0237Gt^ (Fig 5B). Comparisons to published phenotypes of ENU-derived alleles indicated that *fras1* ^mn0156Gt^ and *hmcn1* ^mn0263Gt^ represented novel alleles of known skin blistering mutants *pinfin* (*pif*) and *nagel* (*nel*) [44, 56], respectively (Table 1, Fig 4A–4C).

GBT-tagged loci are revertible due to loxP sites within the RP2 vector (Fig 1A). Cre-mediated recombination permanently excises the mutagenic cassettes, restoring wild-type splicing to the endogenous locus [17]. To verify that *fras1* ^mn0156Gt/mn0156Gt^ and *hmcn1* ^mn0263Gt/mn0263Gt^ insertional mutants were revertible, we tested whether Cre recombinase activity rescued the phenotypes (Fig 1A) [17, 19]. We injected *Cre* mRNA into 1-cell-stage offspring of *fras1* ^+/mn0156Gt^ or *hmcn1* ^+/mn0263Gt^ heterozygote intercrosses, respectively, and scored injected larvae for blistered fins at 48 hpf [44]. The frequency of phenotypic larvae in both *fras1* ^mn0156Gt/mn0156Gt^ (27%, n = 156, Fig 4D) and *hmcn1* ^mn0263Gt/mn0263Gt^ (25%, n = 146, Fig 4E) uninjected controls were within expected Mendelian proportions. However, phenotypic frequencies were significantly reduced in their *Cre*-injected siblings (*fras1* ^mn0156Gt/mn0156Gt^: 5%, n = 140, Fig 4D; *hmcn1* ^mn0263Gt/mn0263Gt^: 8%, n = 223, Fig 4E).

To confirm that our GBT alleles and ENU-induced point mutation alleles of the same locus were functionally equivalent, we conducted complementation testing between *fras1* ^+/mn0156Gt^ and *pif* ^+/tm95b^ [44, 56] heterozygotes. *fras1* ^+/mn0156Gt^ x *pif* ^+/tm95b^ offspring (*fras1* ^+/mn0156Gt^; *pif* ^+/tm95b^ trans-heterozygotes) showed typical *fras1* fin blistering (Fig 4F) in a Mendelian proportion (28%, n = 445), indicating that the *fras1* ^mn0156Gt^ and *pif* ^tm95b^ alleles failed to complement each other. Failure to complement demonstrated that *fras1* ^mn0156Gt^ was a novel, mutagenic allele of the *fras1* locus. Together, the Cre reversion and non-complementation results demonstrated that GBT alleles of known skin mutants phenocopied established ENU mutants.

### *neuregulin 2a* (*nrg2a*) insertional mutants have aberrant apical fin fold morphology

*nrg2a* ^mn0237Gt/mn0237Gt^ homozygotes presented the third recessive phenotype, which was characterized by morphological defects along MFF edges. (Fig 5A and 5B). Whereas wild-type MFF edges are thin and flat (Fig 5A), *nrg2a* ^mn0237Gt/mn0237Gt^ mutant MFF edges were thick and were often accompanied by one or more pointed protrusions along the posterior curvature (Fig 5B). In live embryos, phenotypic changes to MFF edge morphology were visible as early as 48 hpf (Fig 5B). Immunostaining for Collagen II (Col II), an MFF dermal space collagen [42], showed increased Col II accumulation and thick, bent actinotrichia within *nrg2a* ^mn0237Gt/mn0237Gt^ mutant apical MFFs (Fig 5D), whereas actinotrichia in the corresponding distal regions of wild-type MFFs were much thinner or even absent (Fig 5C). In addition, *nrg2a* ^mn0237Gt/mn0237Gt^ mutants’ pectoral fins were often bent or crumpled, or had altered edges similar to those of mutant MFFs (S3A and S3B Fig). Mutant larvae also failed to inflate their swim bladders (S3C and S3D Fig), and died between 8 and 12 days post fertilization (dpf) (S3E Fig). Unlike other published zebrafish skin mutants, however, *nrg2a* ^mn0237Gt/mn0237Gt^ mutants did not display epidermal phenotypes such as cellular aggregates, blistering, or fin fold degeneration during the first 120 hpf [43, 44, 50, 54, 56, 73–76]

To confirm that the GBT insertion caused the MFF phenotype, we conducted Cre rescue experiments on offspring of *nrg2a* ^+/mn0237Gt^ intercrosses. Cre-mediated excision significantly reduced the frequency of phenotypic siblings in injected (7%, n = 322) versus uninjected (25%, n = 358) embryos (p < 0.002) (data not shown). Successful Cre-mediated phenotype rescue confirmed that homozygosity for the *mn0237Gt* allele caused the MFF phenotype.

**Fig 6:**
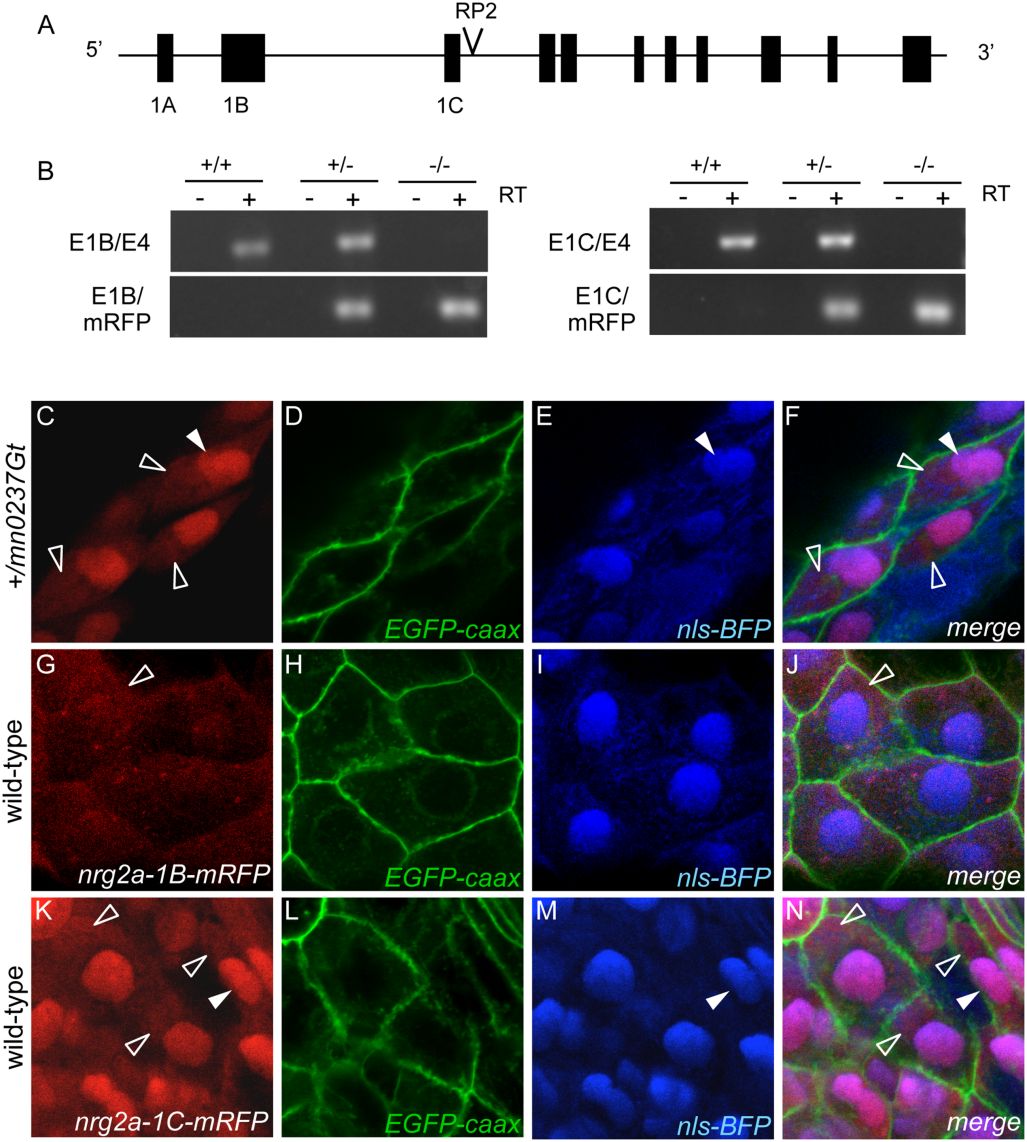
Usage of alternative first exons of *nrg2a* gene leads to differential cytosolic or nuclear localization of resulting protein isoforms. (A) A schematic of the *nrg2a* locus on linkage group 21 (LG21), including alternative first exons 1A, 1B and 1C. The GBT insertion is located in the intron separating exon 1C and exon 2. (B) RT-PCR analyses of 48 hpf sibling embryos from heterozygote (+/*mn0237Gt*) intercross, revealing that endogenous *nrg2a 1B* and *1C* transcripts are only expressed in heterozygous (+/*mn0237Gt*) and wild-type siblings (+/+). *nrg2a 1B-mRFP* and *1C-mRFP* fusion transcripts are only expressed in homozygous mutants (*mn0237Gt*/*mn0237Gt*) and heterozygous siblings (+/*mn0237Gt*). (C-N) Confocal images of MFF epidermis at 24 hpf detecting Nrg2a-RFP fusion protein (C, G, K; red), cell membranes (D, H, L; labeled with EGFP (green) after injection of *egfp-caax* mRNA at 1-cell stage), and cell nuclei (E, I, M; labeled with BFP (blue) after injection of *nls-BFP* mRNA at 1-cell stage). Panels (F, J, N) show merged images. (C-F) +/*mn0237Gt* embryo displays Nrg2a^mn0237Gt^-RFP fusion protein both in the cytoplasm (empty arrowheads) and in the nuclei (filled arrowheads). (G-J) Wild-type embryo injected with mRNA encoding exon 1B-version of the Nrg2a-RFP fusion protein; the fusion protein is largely absent from the nucleus, but present in cyptoplasmic compartments. (K-N) Wild-type embryo injected with mRNA encoding exon 1C-version of the Nrg2a-RFP fusion protein; the fusion protein is present in the cytoplasm and the nuclei, similar to the distribution of the transgene-encoded protein (C-F).

Because the previously reported zebrafish *nrg2a* sequence contained a putative non-coding first exon but did not contain an N-terminal signal sequence [51], we investigated whether additional *nrg2a* N-terminal exons were present. Through 5’RACE and zebrafish genome database searches, we identified two alternative N-terminal exons, exons 1B and 1C. While the putative exon 1A contains only non-coding sequence and is used for the previously reported N-terminally truncated Nrg2a isoforms [51], the newly annotated exons 1B and 1C give rise to alterative N-termini of 145 (1B) or 37 (1C) amino acid residues, respectively (Fig 6A; S2 Fig). Exon 1B has homology to N-termini of *NRG2* homologs in other species, whereas exon 1C shows no homology to any other species. SignalP4 software analysis [77] (http://www.cbs.dtu.dk/services/SignalP/) indicated that only the exon 1B-encoded isoform contains an N-terminal signal sequence. In addition, NucPred software analysis [78] (http://www.sbc.su.se/~maccallr/nucpred/) predicted that the exon 1C-encoded isoform would be capable of nuclear localization (S2 Fig). The gene-breaking cassette that generates the *mn0237Gt* allele is located in the intron between exon 1C and exon 2 (Fig 6A). According to RT-PCR analyses, endogenous *1B* and *1C* transcripts were present in both wild-type (*nrg2a* ^+/+^) and heterozygous (*nrg2a* ^+/mn0237Gt^) siblings, but absent in homozygous mutant (*nrg2a* ^mn0237Gt/mn0237Gt^) siblings (Fig 6B and 6C). Conversely, *1B-mRFP* and *1C-mRFP* fusion transcripts were present in mutant and heterozygous siblings, but absent from wild-type siblings (Fig 6B and 6C). We note that we were unable to amplify the sequence corresponding to non-coding exon 1A [51]. Together, these transcript data indicate that *mn0237Gt* is a null or near-null loss-of-function allele (see also Discussion).

Both *nrg2a*-*mRFP* fusion transcripts (Fig 5G) and Nrg2a^mn0237Gt^–mRFP fusion protein (Fig 5E and 5H) were epidermally localized in 24 hpf *nrg2a* ^+/mn0237Gt^ heterozygotes. WISH analyses using a probe for *mRFP* revealed that the fusion transcript was prominently expressed along the MFF edges (Fig 5E and 5F)—that is, within the apical MFF—and was weakly expressed throughout the entire epidermis (Fig 5G). WISH analyses of endogenous *nrg2a* transcripts in wild-type embryos showed the same pattern (24 hpf; Fig 5H and 5I), thus demonstrating that the *mn0237Gt* insertional allele recapitulates endogenous *nrg2a* expression.

Co-labeled MFF transverse sections further revealed that the Nrg2a^mn0237Gt^–mRFP fusion protein was present in the basal layer of keratinocytes, together with the nuclearly localized basal keratinocyte marker ΔNp63 [32]. In contrast, the EVL, characterized by expression of the *krt4*:*EGFP* transgene [79], lacked Nrg2a^mn0237Gt^–mRFP fusion protein (Fig 5J–5N). Higher magnification analyses confirmed that the punctate distribution of Nrg2a^mn0237Gt^–mRFP fusion protein (Fig 2B; Fig 5E) reflected nuclear localization of the fusion protein (Fig 6C–6F). In addition, Nrg2a^mn0237Gt^–mRFP fusion protein could be detected in the cytoplasm of basal keratinocytes (Fig 5J–5N; Fig 6C–6F). Both of those findings regarding Nrg2a^mn0237Gt^–mRFP subcellular localization were initially surprising because Nrg2a is a growth factor (but see Discussion). However, wild-type embryos injected with synthetic mRNA encoding the exon 1C version of the Nrg2a-mRFP fusion protein showed similar cytoplasmic localization. Exogenous exon 1C-mRFP fusion protein was present in both keratinocyte nuclei and cytoplasm (Fig 6K–6N), mimicking the distribution of the transgene-encoded fusion protein in *nrg2a* ^+/mn0237Gt^ heterozygous embryos (Fig 6C–6F). Exogenous exon 1B-mRFP fusion protein also showed some cytoplasmic localization (Fig 6G–6J). These results were consistent with the presence of one or more putative nuclear localization sequences (NLS) in the predicted amino acid sequence encoded by *nrg2a* exon 1C (S2B Fig), and suggest that the complex subcellular localization of the Nrg2a-mRFP fusion protein is caused by differential distributions of the different N-terminal isoforms.

### *nrg2a* mutants display altered epithelial organization of MFF ridge cells

**Fig 7:**
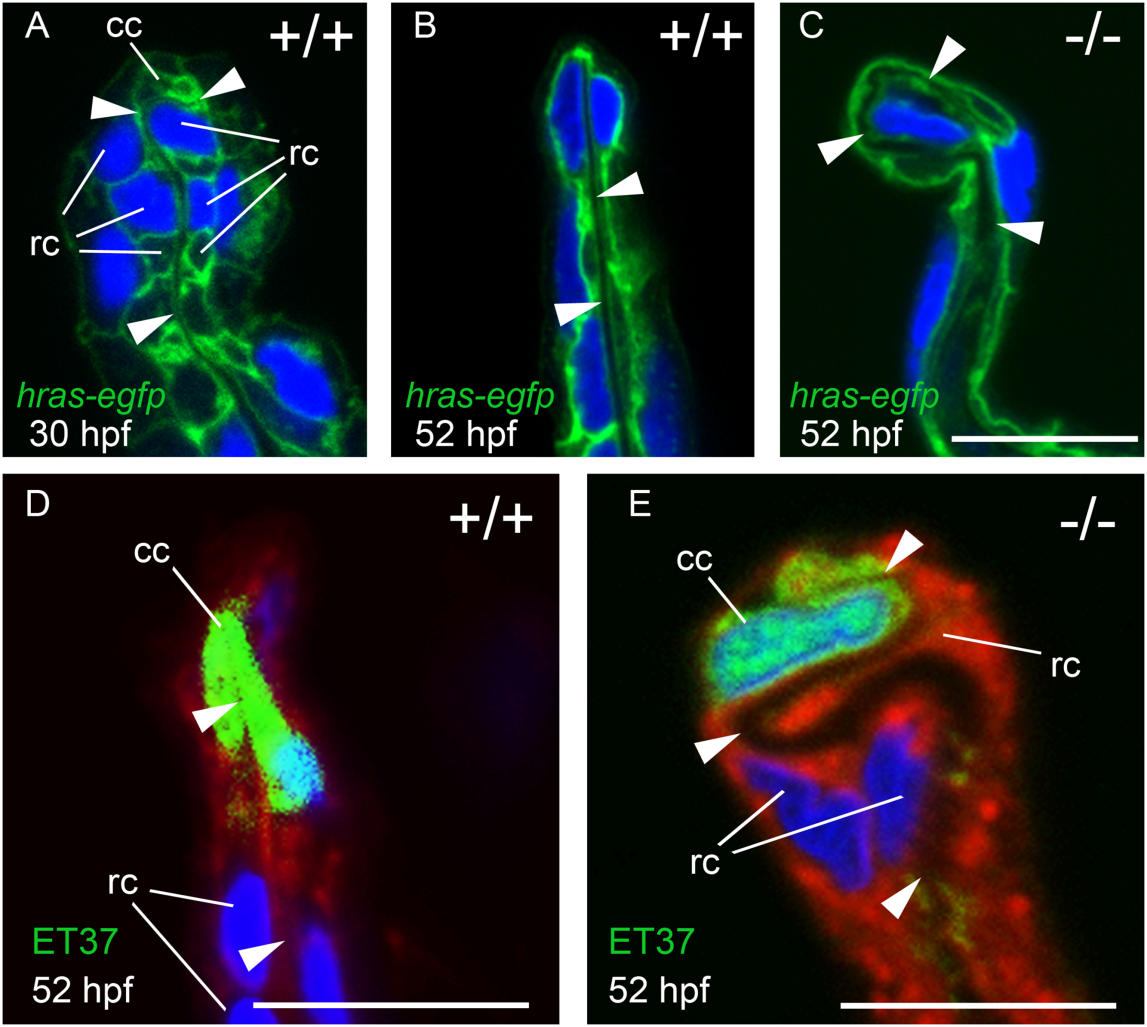
MFF cleft cells of *nrg2a* mutants are largely unaffected, but ridge cells display altered morphology. (A-C) Transverse sections through MFFs of *Tg(Ola*.*Actb*:*Hsa*.*hras-egfp)vu119*-expressing (*hras-egfp*) wild-type and *nrg2a* mutant embryos. Green: membrane-bound EGFP, blue: DAPI. (D-E) ET37 EGFP is expressed in MFF cleft cells (cc). ET37-EGFP: green, CellMask: red, DAPI: blue. (A) At 30 hpf, wild-type ridge cells (rc) are roughly cuboidal, with parallel apical and basal domains. The dermal space (ds) has not yet straightened, especially within the apical MFF terminus bounded by the cleft cell (cc). (B, D) By 52 hpf, the dermal space of wild-type embryos has straightened (arrowheads) and has invaginated into the basal side of the cleft cell. Ridge cells have elongated laterally, adopting a flat, planar, epithelial morphology. Their apical and basal domains are essentially parallel. (C, E) In *nrg2a* mutants, the cleft cell (cc) is present and contains the basal invagination of the dermal space (cleft; E) as in wild-type siblings. In contrast, *nrg2a* mutant ridge cells have elongated incorrectly and display an abnormal morphology, bulging basally into the dermal space, which acquires a serpentine-like appearance. Scale bar: 10 μm. Abbreviations: cc, cleft cell; rc, ridge cell; arrowheads point to dermal space.

**Fig 8:**
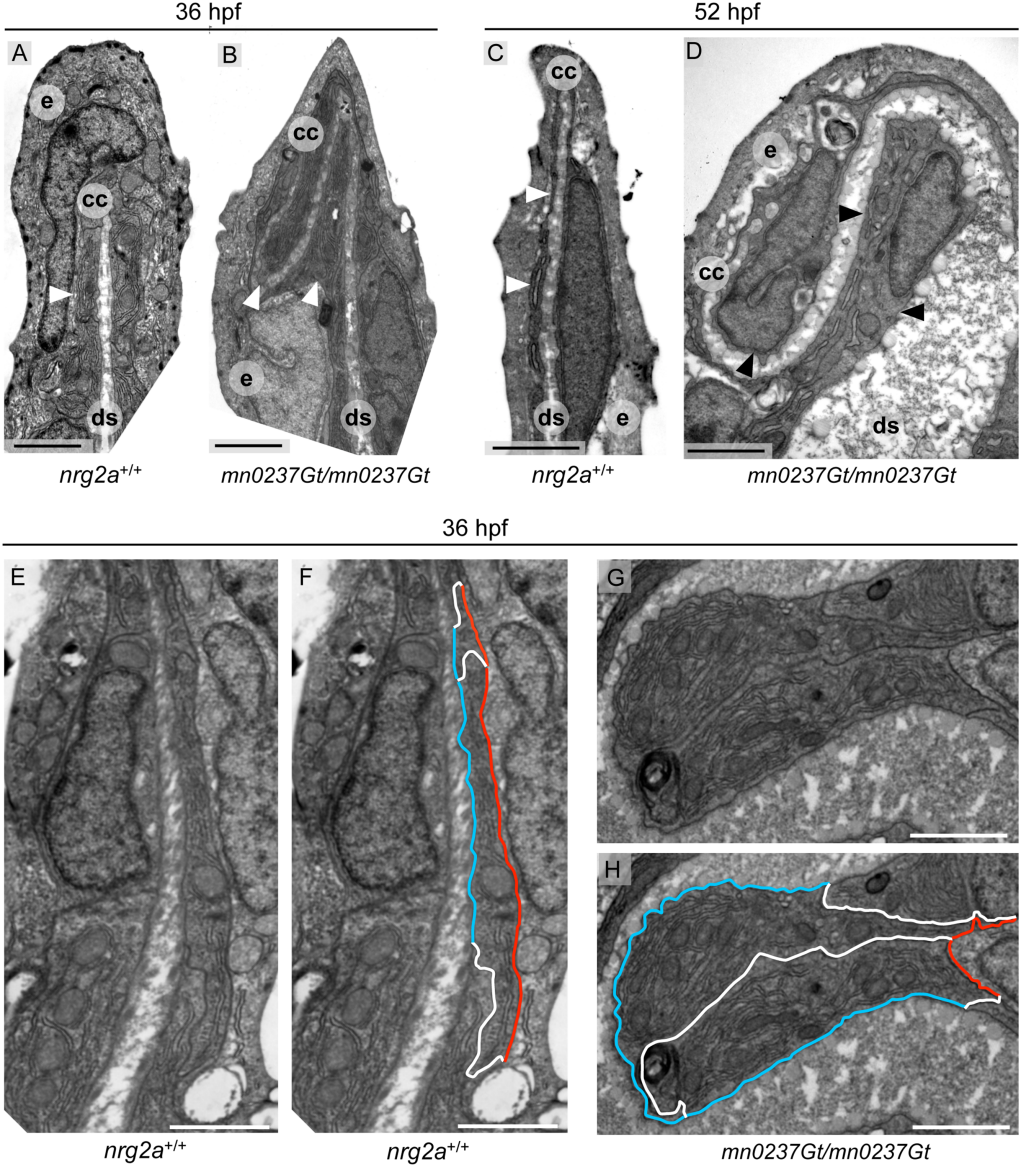
MFF ridge cell in *nrg2a* mutants display alterations in basolateral versus apical dimensions. (A-H) Transmission electron micrographs (TEM) of the distal-most region within wild-type and *nrg2a* mutant MFFs at 36 hpf (A, B, E-H) or 52 hpf (C,D). (A) By 36 hpf, wild-type ridge cells (arrowhead) have begun elongating laterally. Their apical and basal surfaces are parallel to each other and to the dermal space. (C) By 52 hpf, wild-ridge cells have continued elongating and have maintained their arrangements. (B, D) In *nrg2a* mutants (*mn0237Gt*/*mn0237Gt*), ridge cells (arrowheads) are morphologically distorted, fail to stay aligned parallel to the direction of the fin, and bulge into the dermal space, giving it a serpentine-like appearance. (E-H) Relative basolateral and apical dimensions in *nrg2a* mutants are distorted compared to their wild-type counterparts. To illustrate the changes, identical images are shown side by side with and without marked ridge cell borders. (E, F) By 36 hpf, apical (red) and basal (blue) borders of wild-type ridge cells are roughly parallel and of comparable lengths; lateral borders with neighboring basal keratinocytes are in white. (G, H) An example of *mn0237Gt*/*mn0237Gt* mutant ridge cells bulging into the dermal space. The pictured bulge consists of two adjacent ridge cells sharing an exaggeratedly lengthened lateral border (white) and with enlarged basal (blue) borders, but strongly reduced apical borders (red). Scale bars: 2 μm. Abbreviations: ds, dermal space; e, EV; cc, cleft cell; arrowheads point to ridge cells.

To further characterize the MFF defect in *nrg2a* ^mn0237Gt/mn0237Gt^ mutants, we compared transverse sections of median epidermal ridges (MER) from *nrg2a* ^mn0237Gt/mn0237Gt^ mutant larvae with those of wild-type siblings. As described above, the MER consists of a central cleft cell, characterized by a cleft-like invagination of the dermal space, and ridge cells to either side of the cleft cell. To visualize the shapes of individual cleft and ridge cells, we crossed *nrg2a* ^mn0237Gt^ into a *Tg(Ola*.*Actb*:*Hsa*.*hras-egfp)* ^*vu119*^ (*hras-egfp*) ubiquitously expressed membrane-bound EGFP background (Fig 7A–7C). Furthermore, to specifically label cleft cells, we crossed *nrg2a* ^mn0237Gt^ into an ET37 background, in which EGFP is specifically expressed in FMCs and cleft cells (Fig 7D and 7E). We also performed transmission electron microscopy (TEM; Fig 8; S4 Fig) to examine the cleft and ridge cells in finer detail. Because the gross morphological changes characterizing *nrg2a* ^mn0237Gt/mn0237Gt^ mutant MFFs were visible by the second day of development (Fig 5B), we conducted our analyses between 30 and 52 hpf.

During normal MFF morphogenesis, MER cells undergo characteristic consecutive cell shape changes, accompanied by changes in the organization of the developing dermal space between the two apposed epidermal sheets. During the initial steps of fin fold elevation along the body midline, MER ridge cells acquire wedge-like shapes by expanding their lateral and/or apical domains at the expense of the basal domain [38]. But at 30 hpf, re-enlargement of ridge cell basal domains had partially reversed the earlier shape changes. Ridge cells acquired an intermediate shape, more cuboidal than wedge-like, while the dermal space was curved (Fig 7A). MER morphogenesis continued through 36 hpf (Fig 8A, 8E, and 8F; S4A Fig). By 52 hpf, the dermal space had straightened and ridge cells had elongated, acquiring a flattened shape with large, equally-sized apical and basal domains, but small lateral domains (Fig 7B and 7D; Fig 8C).

Cleft cells in *nrg2a* ^mn0237Gt/mn0237Gt^ mutants were only mildly affected. They expressed the ET37 marker (Fig 7E) and retained a typical cleft shape (Fig 7E; S4B Fig), though they often shared an atypically elongated lateral border with a neighboring ridge cell (S4B Fig). Ridge cells in *nrg2a* ^mn0237Gt/mn0237Gt^ mutants, however, displayed far more dramatic alterations (Fig 7C and 7E; Fig 8B, 8D, 8G, and 8H; S4C and S4D Fig). Ridge cells’ lateral domains, and especially their basal domains, were much enlarged, while their apical domains were smaller (Fig 8G and 8H; S4C and S4D Fig), so that they bulged into the dermal space, giving it a serpentine-like appearance (Fig 7C and 7E; Fig 8B, 8D–8G). The bulges consisted either of two ridge cells sharing an extended lateral border (Fig 8G and 8H), or of a single ridge cell with an extended basal domain (S4C and S4D Fig). In both cases, the extent of apical domains was reduced. These findings suggest that Nrg2a regulates ridge cells’ apicobasal organization during apical MFF morphogenesis, thereby promoting or maintaining apical domain identity at the expense of basolateral identity.

To determine whether Nrg2a might have a corresponding function in more proximal (non-MER) epidermal cells of the MFF, we compared 52 hpf and 96 hpf mutant and wild-type siblings via TEM. However, we did not observe consistent epidermal differences, malformations, or cell-cell junction defects in these regions of *nrg2a* ^mn0237Gt/mn0237Gt^ mutants compared to wild-type siblings (data not shown).

### ErbB pathway inhibition phenocopies *nrg2a* mutant MFF defects

**Fig 9:**
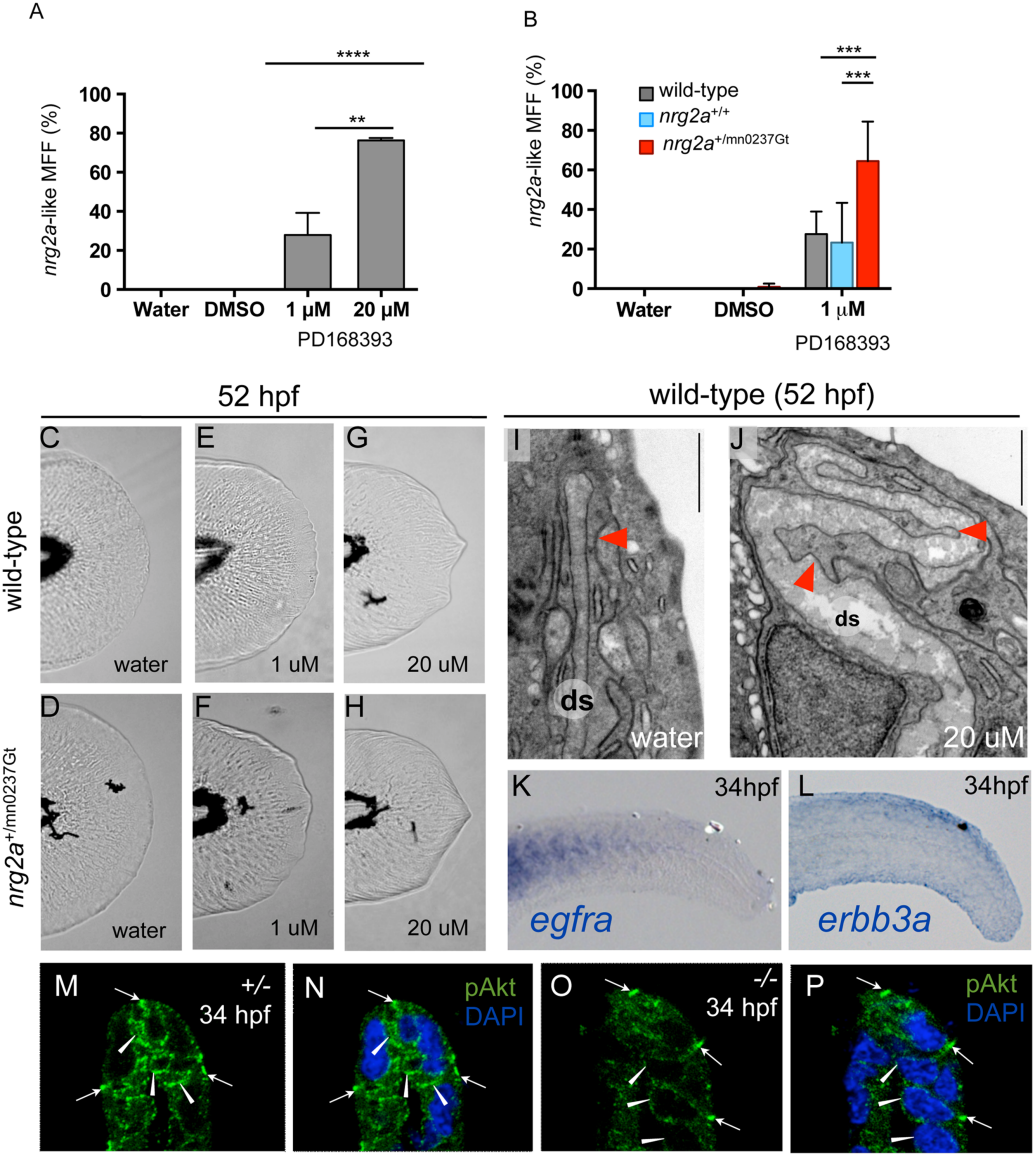
The *nrg2a* MFF phenotype can be mimicked and synergistically enhanced via chemical ErbB inhibition, and is characterized by reduced pAKT levels. (A-J) Pharmacological inhibition of ErbB signaling during MFF morphogenesis (24 through 52 hpf) induces *nrg2a* ^mn0237Gt/mn0237Gt^-like effects in wild-type and, with even higher frequencies, *nrg2a* ^+/mn0237Gt^ heterozygous embryos. (A) MFFs of wild-type embryos treated both with a low dose (1 μM) (28%; n = 120; p = 0.005) or a high dose (20 μM) (76%; n = 102; p < 0.0001) of PD168393 show an *nrg2a* ^mn0237Gt/mn0237Gt^-like MFF morphology at 52 hpf. (B) *nrg2a* ^+/mn0237Gt^ heterozygous embryos (red) are significantly more sensitive to low-dose (1 μM) PD168393 treatment, and display *nrg2a* ^mn0237Gt/mn0237Gt^-like MFF morphology with a higher frequency (64%; n = 152) than treated *nrg2a* ^+/+^ wild-type siblings (blue) (n = 23%; n = 116; p < 0.0001) or treated unrelated wild-type embryos (grey) (28%; n = 120; p < 0.0001). (D-H) Live images of MFFs of representative examples of wild-type (C, E, G) or *nrg2a* ^+/mn0237Gt^ heterozygous (D, F, H) embryos at 52 hpf, after treatment with DMSO (control; C, D), 1 μM PD168393 (E, F) or 20 μM PD168393 (G, H). (I, J) TEM transverse sections of the apical MFF reveal a ridge cell phenotype in PD168393-treated wild-type embryos at 52 hpf. (I) An untreated wild-type embryo has correctly elongated ridge cells (red arrowhead) and a straight dermal space (ds). (J) A sibling embryo treated with 20 μM PD168393 displays basal bulging of MFF ridge cells towards the center the fin fold (red arrowheads) and a corresponding serpentine-like folding of the dermal space (ds), resembling the defects of the *nrg2a* mutant (compare with Fig 8D). (K, L) Whole-mount *in situ* hybridization (WISH) for *egfra* (K) and *erbb3a* (L) in wild-type embryos at 34 hpf (lateral views of tail) reveals epidermally expressed *erbb3a* transcripts (L), whereas *egfra* transcipts are absent in the epidermis, but present in the somites (K). (M-P) Anti-pAKT immunofluorescence of wild-type (M, N) and *nrg2a* mutant (O, P) embryo at 34 hpf; transverse sections through MER region of MFF, counterstained with DAPI (N, P). The wild-type embryo (M, N) displays pAKT localization in distal epidermal MFF cells (ridge cells and cleft cells; arrowheads), whereas pAKT levels in more proximal epidermal MFF cells are much lower. In addition, pAKT is localized at the tight junctions of the outer EVL (arrows), consistent with previously described roles of pAKT to phosphorylate tight junction proteins ZO-1 and Occludin, and to tighten the junctions [122]. In the *nrg2a* mutant (O, P), pAKT signals in ridge and cleft cells are strongly reduced (arrowheads), while pAKT signals at EVL tight junctions are unaltered (arrows).

Because Nrg2 is a ligand for ErbB family tyrosine kinase receptors [80–82], we hypothesized that the lack of Nrg2a-mediated ErbB signaling in *nrg2a* ^mn0237Gt/mn0237Gt^ mutants caused the ridge cell phenotype. We tested whether pharmacological ErbB inhibition in wild-type embryos would phenocopy *nrg2a* ^mn0237Gt/mn0237Gt^ mutants. Because serpentined distal dermal spaces were observed as early as 36 hpf (Fig 8A), we incubated wild-type embryos in either a low dose (1μM) or high dose (20μM) of the small-molecule EGFR/pan-ErbB inhibitor PD168393 [52, 83] from 24 hpf through 52 hpf. We examined MFF morphology in live embryos at 52 hpf (Fig 9C–9G) and 96 hpf (Fig 9A), and via TEM at 52 hpf (Fig 7I and 7J).

At 52 hpf and 96 hpf, PD168393-treated wild-type larvae displayed thickened MFF edges (Fig 9A, 9E, and 9G), similar to those observed in *nrg2a* ^mn0237Gt/mn0237Gt^ mutants (Fig 5B). The response was dose-dependent (Fig 9A, 9E, and 9G). TEM analyses of randomly selected 20μM PD168393-treated embryos (n = 21) showed serpentine-like MFF distal dermal spaces (Fig 9J) indistinguishable from distal dermal spaces in *nrg2a* insertional mutants (compare with Fig 8D). These data indicate that inhibiting ErbB receptor signaling in wild-type embryos recapitulates the *nrg2a* ^mn0237Gt/mn0237Gt^ phenotype.

### *nrg2a* ^+/mn0237Gt^ heterozygotes are sensitized to ErbB inhibition

Because our data point to a functional connection between Nrg2a and ErbB signaling, we conducted a genetic interaction analysis to test whether partial loss of both Nrg2a and ErbB signaling had synergistic effects on MFF morphogenesis. Specifically, we tested whether *nrg2a* ^+/mn0237Gt^ heterozygous larvae were more sensitive to moderate ErbB inhibition than were their wild-type siblings. In fact, significantly more *nrg2a* ^+/mn0237Gt^ heterozygotes (64%, n = 152, p < 0.0001) phenocopied *nrg2a* ^mn0237Gt/mn0237Gt^ mutants at the low PD168393 dose (1 μM) than did sibling (23%, n = 116) or non-sibling (28%, n = 120) wild-type embryos (Fig 9B; compare also Fig 9E with Fig 9F). In contrast, *nrg2a* ^+/mn0237Gt^ heterozygosity did not further enhance the phenotype caused by the high (20 μM) PD168393 dose (compare Fig 9G with Fig 9H). Together with our earlier inhibitor data, these studies indicate that the *nrg2a* ^mn0237Gt/mn0237Gt^ MFF phenotype results from a loss of Nrg2a-mediated ErbB signaling.

### MFF epidermis displays *erbb3a* expression and AKT phosphorylation, which is reduced in *nrg2a* mutants

Vertebrates have four different Neuregulins (NRG1-4) and four different members of the epidermal growth factor (EGF) receptor ErbB family of receptor tyrosine kinases (EGFR/ErbB1/HER1, ErbB2/Neu/HER2, ErbB3/HER3, and ErbB4/HER4) [53]. The combinatorial possibilities of those ligands and receptors permit diversity and specificity in signaling. Just as EGF binds to and signals through ErbB1/ErbB1 homodimers or ErbB1/ErbB2 heterodimers, NRG1 and NRG2 preferentially signal via ErbB3/ErbB2 heterodimers, while NRG3 and NRG4 do so via ErbB4 [53, 80–82, 84]. Intracellular signal transduction of all ErbB receptors can occur through mitogen-activated protein kinase (MAPK) pathways, although p85-mediated activation of the PI3K –AKT pathway has been described as the major signal transduction route for ErbB3 in particular [53, 85–88]. Consistent with the requirement for Nrg2a during zebrafish apical MFF morphogenesis, we observed strong epidermal expression of its potential co-receptor *erbb3a* during the 30 to 36 hpf developmental window (Fig 9L; 34 hpf). However, we failed to detect epidermal expression of the zebrafish EGF receptor gene *egfra (erbb1a)* during the same timeframe (Fig 9K; 34 hpf). Furthermore, and consistent with possible ErbB3 involvement, immunofluoresence analyses revealed phosphorylated (activated) AKT (pAKT) in MFF ridge cells at 34 hpf (Fig 9M and 9N). Strikingly, pAKT levels were strongly reduced in *nrg2a* ^mn0237Gt/mn0237Gt^ mutants’ MFF ridge cells, but not at other sites (Fig 9O and 9P). Unlike the dramatic changes in pAKT levels, activated ERK (pERK) levels in *nrg2a* ^mn0237Gt/mn0237Gt^ mutants did not change relative to pERK levels in wild-type siblings (S5 Fig). Together, these data suggest that Nrg2a regulates MFF ridge cell apicobasal organization and morphogenesis via ErbB3-mediated PI3K –AKT signaling.

### Concomitant loss of Lgl2 function alleviates *nrg2a* mutant MFF defects

**Fig 10:**
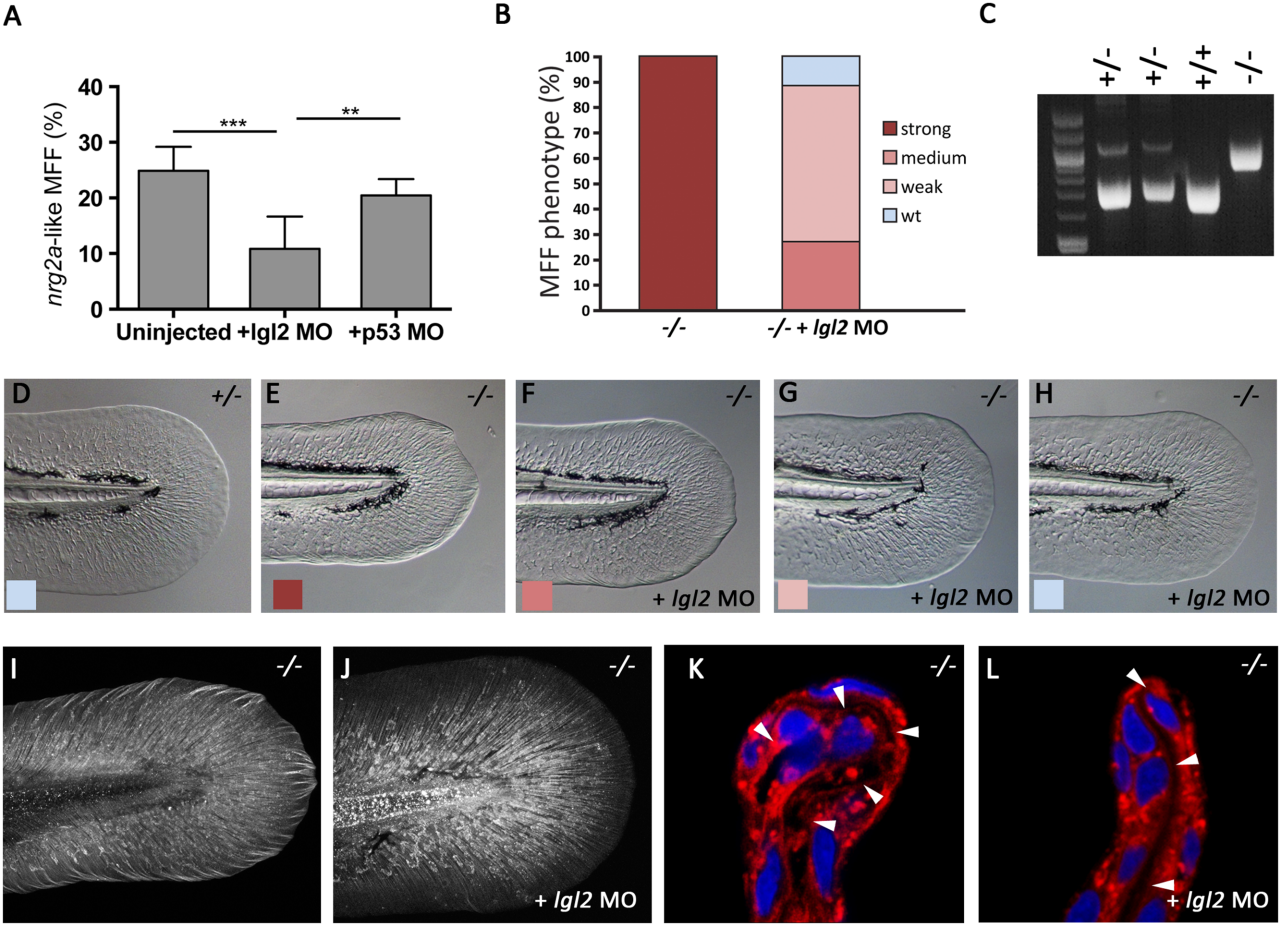
The MFF phenotype of *nrg2a* mutants is rescued upon concomitant loss of Lgl2 function. (A) At 52 hpf, morpholino (MO)-mediated knockdown of *lgl2* significantly ameliorated the *nrg2a* mutant phenotype (12%, n = 639, p < 0.0001) relative to uninjected embryos of an *nrg2a* -/+ intercross (25%, n = 943). Percentages represent the mean of means (MOM); error bars represent the standard deviations (SD). (B) Percentages of genotyped *nrg2a*-/- mutants with a strong, medium, weak, or wild-type MFF phenotype, classified by morphological criteria at 52 hpf. While uninjected *nrg2a* mutants (n = 20) all display a strong phenotype, *lgl2* MO-injected mutants (n = 26) show medium, weak or no MFF defects. (C) PCR products obtained via *nrg2a* genotyping of representative *nrg2a* -/+, +/+ and-/- embryos at 52 hpf (see Materials and Methods). (D-H) Tail fins of representative live embryos at 52 hpf, as used for quantitative classification in panel B: wild-type (D), uninjected *nrg2a*-/- mutant with strong MFF phenotype (E), and *lgl2* MO-injected *nrg2a* -/- mutant embryos with medium (F), weak (G) or wild-type (H) phenotype. (I, J) Tail fins of genotyped uninjected (I) and *lgl2* MO-injected (J) *nrg2a* -/- mutant embryo at 48 hpf. Col II immunostaining reveals a normalized organization of collagenous actinotrichia within the dermal space of the Nrg2a/Lgl2-double-deficient embryo (J; compare with Fig 5C for wild-type condition). (K, L) Transverse sections through the dorsal MFF of a genotyped uninjected (K) and an *lgl2* MO-injected (L) *nrg2a* -/- mutant embryo at 52 hpf; CellMask (red) and DAPI (blue) staining reveals a rescue of the dermal space (indicated by arrowheads) from a serpentine-like organization (K) to a straight organization (L) in the Nrg2a/Lgl2-double-deficient embryo (L; compare with Fig 7D for wild-type condition).

To date, ErbB activity in zebrafish epidermis has only been described in the context of the tumor suppressor *lethal giant larvae 2* (*lgl2*), which blocks ErbB signaling and epithelial-to-mesenchymal transitions (EMT) to safeguard epidermal integrity during late larval stages (120 hpf) [52]. Yet even though *lgl2* is epidermally expressed by 24 hpf [54], it has no identified developmental role during that early time frame. However, previous characterizations of Lgl2 as an epithelial polarity regulator that promotes basal fate [54, 89, 90], along with the basolateral domain expansion observed in *nrg2a* ^mn0237Gt/mn0237Gt^ mutants’ MFF ridge cells and the temporal overlap among *lgl2* [54], *nrg2a* (Fig 5H and 5I), and *erbb3a* expression patterns (Fig 9L), led us to speculate that Lgl2 might play an earlier role in epidermal development. We hypothesized that that earlier Lgl2 role involved opposing Nrg2 –ErbB3 signaling during MFF morphogenesis. To test that hypothesis, we suppressed *lgl2* in the *nrg2a* ^mn0237Gt/mn0237Gt^ background. Morpholino (MO) knockdown [11] of *lgl2* significantly restored *nrg2a* ^mn0237Gt/mn0237Gt^ mutants’ MFF morphology at 52 hpf (Fig 10A–10H) relative to both uninjected controls and *tp53* MO-injected controls (Fig 10A). Indeed, *lgl2* MO-injected and genotyped *nrg2a* ^mn0237Gt/mn0237Gt^ embryos displayed external MFF morphology indistinguishable from that of wild-type siblings (compare Fig 10H with Fig 10D). Furthermore, MFF internal organization was also ameliorated in *lgl2* MO-injected *nrg2a* ^mn0237Gt/mn0237Gt^ embryos. Dermal actinotrichia organization, (Fig 10I and 10J; 48 hpf), ridge cells’ apicobasal organization, and MFF dermal space bending (Fig 10K and 10L; 52 hpf) were all dramatically normalized, leading to embryos indistinguishable from wild-type (compare Fig 10J with Fig 5C, and Fig 10L with Fig 7D). These results indicated that loss of Lgl2 activity suppressed the *nrg2a* phenotype, revealing a new and significantly earlier role for *lgl2* in fin fold development, distinct from its established tumor-suppressing and hemidesomosome (basal)-promoting functions in the later body epidermis [52, 54].

## Discussion

### GBT mutagenesis further develops the zebrafish as a skin biology model

By selecting for skin- and MFF-localized mRFP, we identified four genes with little or no previously appreciated connection to skin biology: *megf6a*, *nrg2a*, *arhgef25b*, and *fkbp10b* (Fig 1B and 1B’; Fig 2A, 2B, 2E, 2F, 2K and 2L). In addition, we isolated GBT alleles of several known skin-related genes (Table 1; Fig 1C–1E’; Fig 2). In zebrafish embryos, apical MFF basal keratinocytes express *fras1*, *grip1*, and *hmcn1* [44, 45], and MFF mesenchymal cells (FMCs) express *msxc* [39]. *ahnak* and *capn12* have not yet been investigated in zebrafish, but have been detected in mammalian stratified squamous epithelia [70, 91]. *col4a4* encodes one (A4) of the six possible constituents (A1-A6) of collagen IV, which forms large networks integral to BMs. Zebrafish *col4a4* expression has thus far only been analyzed via RT-PCR of whole-body RNA extracts, without corresponding spatial resolution [8]. However, in humans, cutaneous collagen IV largely employs subunits other than COL4A4 [67], and *COL4A4* loss-of-function mutations, such as Alport Sydrome, largely affect epithelia other than the skin [92]. Thus, zebrafish *col4a4* ^mn0275Gt^ cutaneous expression offers a new tool for investigating evolutionary changes in tissue-specific expression of these important BM components and the impact of those changes on the biology of specific organs.

With *fras1*, *grip1*, and *hmcn1*, we identified GBT alleles of loci with documented functions in zebrafish MFF development and connections to human skin disease. In humans and mice, recessive loss-of-function mutations in *FRAS1* and *GRIP1* cause the rare, clinically overlapping congenital disorders Fraser Syndrome and Ablepharon Macrostomia Syndrome [47, 48, 64, 65]. Both are characterized by malformations resulting from epidermal or epithelial blistering during fetal development due to compromised anchorage of epidermal and other epithelial basement membranes to underlying connective tissues such as the dermis. *hmcn1* has recently been proposed as an additional Fraser Syndrome gene, though that hypothesis awaits confirmation [44]. *fras1* ^mn0156Gt/mn0156Gt^ and *hmcn1* ^mn0263Gt/mn0263Gt^ homozygotes fully phenocopied the respective ENU-induced mutants [44] (Fig 4A–4C). Consistent with previous MO knockdown results, *grip1* ^mn0078Gt/mn0078Gt^ homozygotes lacked an overt skin phenotype, likely due to partial functional redundancy with zebrafish *grip2* [44].

In contrast with the ENU-induced alleles, however, the *fras1* ^mn0156Gt^ and *hmcn1* ^mn0263Gt^ GBT alleles are revertible in a Cre-dependent manner (Fig 4D and 4E). That reversion capability offers important experimental advantages. For instance, coupling transgenic methods for temporally and/or spatially restricted Cre expression with GBT reversion would allow investigators to parse more finely the spatiotemporal requirements for Fras1 and Hmcn1 in MFF development. Similarly, tissue-specific Cre expression could be used to rescue the lethal craniofacial defects of *fras1* mutants [93], thereby permitting investigations into possible later defects in *fras1* mutants, including possible later consequences of the (non-lethal) epidermal blistering. In mammals, transient epidermal blistering in Fras1 mutant embryos is thought to abrogate crucial epithelial—mesenchymal interactions, leading to characteristic later defects such as syndactyly (fused digits) or cryptophthalmos (fused eye lids) [47–49]. Thus far, it has not been possible to investigate possible later consequences of embryonic epidermal blistering in the zebrafish model due to the lethal craniofacial defects. But as outlined above, such studies could now be carried out with the new revertible GBT allele.

The 11 ZIP lines described here (Table 1; Fig 1B–1E’; Fig 2) comprise 3% of the original 350 GBT lines [17]. Continued expression cataloging and gene identification will build a comprehensive, *in vivo* spatiotemporal anatomical expression atlas for integument development and diseases, revealing relationships among anatomy, gene expression, and protein localization. That unique resource would be a valuable supplement to standard anatomical atlases [94, 95] as well as a comparative resource for mammalian skin biology studies.

### The *nrg2a* ^mn0237Gt^ allele

Of the 11 identified loci, 3 gave a morphologically visible larval skin phenotype when their respective gene-breaking alleles were bred to homozygosity (*fras1*, *hmcn1*, *nrg2a*). The question of whether any of the other identified loci are required during later stages of skin biology requires further investigation since our analyses only extended through 120 hpf. Because the *fras1* and *hmcn1* mutant phenotypes had already been characterized, we focused on the *nrg2a* mutants, which displayed specific defects during MFF morphogenesis.

As with the other Neuregulin family members [96], zebrafish *nrg2a* exists in multiple isoforms due to differential promoter usage and alternative splicing. Previously reported isoforms [51] would have lacked the N-terminal sequences described here because exon 1A is a non-coding exon. Such 1A isoforms would therefore lack the first 145 amino acid residues of the 1B isoform and the first 37 N-terminal amino acid residues of the 1C isoform that we describe here (S2 Fig). We successfully amplified 1B and 1C transcripts from 5’ RACE and RT-PCR analyses, but were unable to successfully amplify the 1A isoform. Those results suggest that the 1B and 1C versions are the predominant *nrg2a* transcripts generated during the stages relevant to MFF development. It should also be noted that Honjo *et al*. used MOs targeting an internal splice site present in all isoforms, rather than the 1A-specific translational start region, leaving open the question of which isoform(s) are required for dorsal root ganglia development [51].

Most important for our present work, however, is the fact that all three *nrg2a* alternative first exons, 1A-C, are located upstream of the GBT insertion site that generates the *nrg2a* ^mn0237Gt^ allele. Consequently, *nrg2a* ^mn0237Gt/mn0237Gt^ mutants have truncated *nrg2a* transcripts which lack the sequences encoded by exon 2 onwards due to the GBT cassette’s transcription termination sequence (Fig 1A). Accordingly, the 1B and 1C transcripts give rise to Nrg2a-mRFP fusion proteins in which the N-terminal 138 or 29 amino acids encoded by exons 1B or 1C, respectively, are directly fused to mRFP. 1A transcripts are not translated at all because the endogenous translational start codon is localized in exon 2. Therefore, *nrg2a* ^mn0237Gt^ is most likely a null or near-null loss of function allele.

It is also noteworthy that out of all three N-terminal isoforms, only the longer and phylogenetically conserved 1B isoforms contain an N-terminal signal sequence involved in co-translational protein translocation to the endoplasmatic reticulum and subsequent secretion [97]. However, all endogenous isoforms share an internal transmembrane domain [51] which can target the proteins to the cell membrane [98] from where their biologically active ectodomains can be released via proteolytic processing [96]. Nrg2a^mn0237Gt^-mRFP fusion proteins lack that internal transmembrane domain because they only contain exon 1-encoded Nrg2 sequences. The absence of that domain in Nrg2a^mn0237Gt^-mRFP fusion proteins may contribute to the cytoplasmic and nuclear localization of the 1C fusion protein, which also lacks an N-terminal signal sequence, but instead contains at least one nuclear localization sequence (S2 Fig). This suggests that the unexpected distribution of the fusion proteins might be a special feature of the targeted locus and a consequence of the lack of crucial internal domains in the encoded proteins, thus demonstrating an expected limitation (loss of protein trafficking signals due to truncation) of GBT technology to recapitulate the subcellular distribution of the endogeneous protein counterparts. On the other hand, we cannot rule out that the RFP localization observed in *nrg2a* ^mn0237Gt^ embryos does reflect the actual subcellular distribution of the endogenous Nrg2 proteins. Indeed, nuclear localization has also been reported for several other RTK ligands, such as different Fibroblast growth factors (FGFs) and the Neuregulin relative EGF, as well as their receptors [99–103]. Studies with isoform-specific Nrg2a antibodies or full-length Nrg2-RFP fusion constructs will be needed to give more definitive answers.

### Nrg2a/ErbB3 signaling is an essential regulator of ridge cells’ apicobasal organisation and MFF morphogenesis

In mice, loss of Nrg2 leads to growth retardation and reduced reproductive capacity [104]. We were unable to address whether the loss of Nrg2a in zebrafish has similar consequences because *nrg2a* ^mn0237Gt/mn0237Gt^ mutants die during larval stages, well before the onset of significant somatic growth and sex differentiation. The reason for this larval death is currently unknown. However, the defects during MFF morphogenesis described here are most likely not the primary cause, because other mutants can survive in the complete absense of median fins (M.H., unpublished observations).

Altered MFF morphology similar to that of *nrg2a* ^mn0237Gt/mn0237Gt^ mutants has only been reported for one other mutant to date—*erbb2* ^-/-^, which harbors a loss-of-function mutation in EGF/NRG co-receptor ErbB2. However, the MFF morphological defects of *erbb2* ^-/-^ mutants have not yet been analyzed in additional detail [52]. The initial steps of MFF formation and elevation involve basal detachment of midline keratinocytes and their transition from a cuboidal to a wedged morphology, which is marked by shrinkage of their basal domains [37, 38]. The distal-most basal keratinocyte in the tip of the elevating fold becomes the cleft cell; the two to three basal keratinocytes adjacent to each cleft cell become ridge cells. As we have documented here, ridge cells then transition a second time, re-elongating their basal domains at the expense of their apical domains to reverse their wedged morphology and return to their initial rectangular/cuboidal morphology (Fig 6A). Finally, ridge cells then become progressively flatter by extending their basal and apical sides at the expense of their lateral domains (Fig 6F and 6H). This second phase of cell shape changes is affected in *nrg2a* mutants: *nrg2a* mutant ridge cells display disproportionately expanded basolateral domains and bulge basally, deforming the dermal space into serpentine shapes (Fig 6C, 6E, 6G and 6I; S3C–S3F Fig). In addition, ectopic and enlarged actinotrichia in the distal-most regions of the MFF (Fig 5H) suggested increased basal activity such as secreting ECM components into the dermal space. Together, these findings suggest that coordinating apical and basal extension during ridge cells’ second shape change requires active Nrg2a –ErbB signaling which plays a “pro-apical” role during that transition by promoting maintenance of the apical domain and/or antagonizing basolateral epithelial domains to counterbalance the “basalization” of ridge cells that occurs during their transition from a wedged back to a rectangular shape. These shape changes during the second phase of MFF morphogenesis mainly occur in the distal-most MFF cells (ridge and cleft cells), whereas more proximal MFF basal keratinocytes remain rectangular during their initial recruitment into the MFF [37]. Accordingly, later MFF morphogenetic steps require less- pronounced shape changes by those proximal basal keratinocytes, which could explain why they are not affected in *nrg2a* mutants. Consistent with the spatially restricted defects in the mutant, distal MFFs in wild-type embryos show stronger endogenous *nrg2a* expression (Fig 5H and 5I). Ridge cells’ higher pAKT levels (Fig 9M–9P) also suggest that the apical MFF receives stronger Nrg2a signaling than do proximal MFF cells. In contrast, expression of the likely Nrg2a receptor gene *erbb3a* is fairly homogeneous throughout all basal epidermal cells (Fig 9K). In conclusion, we have good evidence that the distal restriction of the defects in the mutants is due to spatially restricted signaling in wild type embryos. Nevertheless, it remains unclear why only the ridge cells, but not the cleft cells, are affected in *nrg2a* mutants.

### Nrg2a –ErbB signaling and Lgl2 display antagonistic effects during the separate processes of epidermal morphogenesis and homeostasis

Identifying the molecular and cellular mechanisms underlying the pro-apical and/or anti-basal effects of Nrg2a-ErbB signaling will require additional investigation. The expression of *erbb3a*, but not *egfra*/*erbb1*, at the appropriate locations (Fig 9), together with previously reported *erbb2* epidermal expression in zebrafish embryos and the requirement of *erbb2-/-* for proper MFF morphogenesis [52], suggests that Nrg2a may signal via Erbb2/3 receptor heterodimers, which would be consistent with biochemical findings [53]. In addition, reduced pAKT levels, but normal pERK levels in *nrg2a* mutant MFF keratinocytes (Fig 9; S5 Fig) points to Nrg2a –ErbB signaling via the PI3K –AKT signal transduction pathway, rather than the MAPK/ERK pathway, as has also found for Neuregulin signaling in other instances [53, 85, 87, 88].

Our finding that concurrent loss of Lgl2 activity significantly alleviated MFF defects in *nrg2a* mutants suggests that Nrg2a’s pro-apical effects extend to antagonizing Lgl2 pro-basal activity. As a homolog of the *Drosophila* cell polarity gene *lethal giant larvae* (*lgl*), *lgl2* is a cell polarity regulator required for maintaining the basolateral domain in epithelial cells [89, 90]. Likewise, the epidermal defects of zebrafish *lgl2* mutants can also be interpreted in terms of loss of basolateral identity. Basal keratinocytes in *lgl2* mutants not only lack hemidesomomes, basal domain junctions involved in attachment to underlying BM [54], but also display an even more pronounced loss of basal characteristics by undergoing EMT. Such events point to a tumor-suppressor role for Lgl2 [52]. Overactive ErbB signaling and its PI3K –AKT signal transduction branch, on the other hand, have well-known oncogenic effects, promoting EMT and hyperproliferation [105–107]. Thus, the roles of the Nrg2a –ErbB3 –AKT axis and Lgl2 during MFF morphogenesis identified in this study suggest that in addition to the established antagonistic relationship between ErbB signaling and Lgl2 during later phases of epidermal homeostasis [52] and carcinogenesis, the Nrg2a –ErbB3 –AKT signaling axis and Lgl2 have as-yet unappreciated antagonistic, and more moderate anti- or pro-basal effects, respectively, on keratinocytes during earlier steps of epidermal morphogenesis. To our knowledge, our data also provide the first indication that AKT/pAKT is involved in regulating apicobasal organization and epithelial cell polarity during the development of any species.

Strikingly however, despite their early and late antagonisms, Lgl2 and Nrg2a *per se* are uniquely required during different stages of epidermal morphogenesis and homeostasis. Lgl2 is required during late stages: epidermal defects in *lgl2* mutants only become apparent between 108 and 120 hpf [52], and MFF formation is unaffected. In contrast, Nrg2a is only required early: mutants develop MFF defects between 30 and 36 hpf (Fig 6), and we did not detect any later-stage body epidermis defects corresponding to those of *lgl2* mutants (data not shown). Yet concomitant loss of Lgl2 rescues the early MFF defects of *nrg2* mutants (Fig 10), while concomitant loss of ErbB2 signaling rescues the late defects of *lgl2* mutants [52]. Genetically, these findings argue that Lgl2 is epistatic to Nrg2a –ErbB signaling during early MFF morphogenesis: in double-deficient zebrafish embryos, the Lgl2 phenotype (no MFF defects) was dominant over the *nrg2a* phenotype. This suggests that Nrg2a –ErbB acts at least partly by blocking Lgl2 activity, and that the MFF phenotype of Nrg2a-deficient embryos is at least partially caused by Lgl2 overactivity. That proposition fits with previous findings that EGF treatment suppresses *LGL2* transcription in human cell culture systems [108], but confirming it will require analyzing Lgl2 protein levels in *nrg2a* mutant embryos [108]. ErbB signaling, on the other hand, appears to be epistatic to Lgl2 during later epidermal homeostasis and carcinogenesis: in ErbB2/Lgl2-double deficient zebrafish larvae, the *erbb2* phenotype (no EMT, no keratinocyte hyperproliferation) was dominant over the *lgl2* phenotype [52]. Those findings, together with increased levels of ErbB signaling mediator pERK in *lgl2* mutants, had led to the conclusion that Lgl2 acts by blocking ErbB signaling, and that the MFF phenotype of Lgl2-deficient embryos is at least partially caused by ErbB overactivity. Together, our data and those of Reischauer *et al*. [52] suggest the existence of a mutual negative feedback mechanism in which Lgl2 blocks ErbB signaling and vice versa. However, these opposing interactions occur at different developmental stages, and may involve different ErbB signaling molecules. Thus, early MFF ridge cell morphogenesis involves Nrg2a, most likely acting through ErbB2/3 and the PI3K –AKT signal transduction pathway (Fig 9), whereas later carcinogenesis involves the ERK MAPK signal transduction pathway [52], most likely activated by EGF and ErbB1/2.

### Neuregulins may also be involved in mammalian epithelial morphogenesis and carcinogenesis

Although studies of Neuregulins have primarily addressed their roles during neuronal or neural crest development [109, 110], expression of the NRG ligand family has also been described in epithelial contexts, most notably in mammary gland epithelium. Neuregulins and their ErbB receptors are expressed in murine embryonic mammary gland epithelium [111]. Expression of Neuregulins 1, 2, 3, and 4 has also been reported in breast cancer cell lines [112] and ductal carcinomas [113]. There is also some evidence that NRG2 has an epidermal role. A transcriptional profiling study of differential gene expression during human epidermal differentiation found that *NRG2* was expressed in basal keratinocytes, while suprabasal cells expressed the known *NRG2* receptor *ERBB3* and its likely co-receptor *ERBB2* [72]. In light of these findings, our data regarding the role of Nrg2a during zebrafish MFF development may also provide new insights into regulation of epithelial polarity and morphogenesis during mammalian epithelial development and carcinogenesis. Overall, GBT technology provides a valuable leap forward in using the vertebrate zebrafish model to identify important molecular players and to gain new insights into the genetic control of skin biology and disease.

## Materials and Methods

### Larval and adult care and maintenance

Adult fish and embryo care was performed according to standard protocols approved by the on-site Institutional and Public Animal Care and Use Committees. Wild-type stocks were derived from the offspring of adult zebrafish obtained from Segrest Farms (Segrest Farms, Florida, USA). Non-GBT transgenic marker lines used were enhancer trap ET37 [46, 60], ubiquitously expressed membrane-bound EGFP *Tg(Ola*.*Actb*:*Hsa*.*hras-egfp)* ^*vu119*^ [75, 114] and *Tg(krt4*:*GFP)* ^*gz7*^ [79].

### *in vivo* imaging of live larvae

Live larvae were imaged for mRFP fluorescence on an Axio ImagerZ.1 ApoTome microscope (Zeiss) with AxioVision software (Zeiss). Z-stack images were flattened using the AxioVision Multi-Image Projection (MIP) tool. SCORE imaging techniques [115] were used to hold live larvae and optimized for high-quality fluorescent imaging as follows: live larvae were transferred into 2.5–2.6% methylcellulose and drawn into borosilicate glass capillaries (World Precision Instruments WPI #1B120-3). 80% glycerol was used as the imaging medium between capillary and cover slip. Brightfield images of live larvae were obtained either by mounting the specimen in 1.5% methylcellulose and imaging with a Zeiss Axiophot microscope using the Zeiss AxionCam HRc camera and Axiovision software, or by SCORE imaging on a Zeiss Axioplan2 microscope using a Canon PowerShot A640 camera with Remote Capture Task and Canon Image Browser software.

### Cre mRNA phenotype rescue

Cre-mediated excision of GBT mutagenic cassettes was performed as previously described [17]. *fras1* and *hmcn1* reversion experiments were scored at 48 hpf; *nrg2a* reversion experiments were scored at 96 hpf. Results were graphed with PRISM software (Graphpad); *p*-value and significance were calculated with *t*-tests.

### GBT allele identification

Candidate loci for ZIP GBT insertions were identified from fusion transcripts using 5’RACE on race-ready cDNA (RR-cDNA) as previously described [17] or from genomic DNA (gDNA) using TAIL PCR [116, 117]. Candidate insertion loci were confirmed or rejected by genotyping 8 individual sibling larvae, 4 mRFP^+^ and 4 mRFP^-^, for a correctly oriented RP2 insertion within the candidate locus. Individual genotyping reactions were designed to identify the genomic-transposon junction (“junction product”) within a specific locus of interest using one transposon-specific primer and one gene-specific (GS) primer. A candidate insertion locus was confirmed if all mRFP^+^ siblings were positive for the junction product within that locus of interest and all mRFP^-^ siblings were negative for that same junction product. The transposon-specific primers were *5R-mRFP-P2* (5’-CCTTGAAGCGCATGAACTCCTTGAT-3’) (used with all forward (“F”) GS primers) and *3R-GM-P2* (5’-TGGGATTACACATGGCATGGATGAAC-3’) (used with all reverse (“R”) GS primers). GS primers were as follows: *GNT-SEC0078-F3* (5’-CAAGGCACAGCAAGGCTGGTGTA-3’), *GNT-SEC0078-R0* (5’-CGGACAGATTTTCACTCATACATTGCCT-3’), *RT-SEC0156-F1* (5’-CTGCTCAAAGGCTCTGATCTTGAACC-3’), *RT-SEC0156-R1* (5’-ccacacagaccctacaggacgaatg-3’), *RT-SEC0175-F1* (5’-GCTGTTCACCCTTCTGTTGGAAGGTT-3’), *RT-GBT0175-R1* (5’-AGTGGAGACAGAGGCTGAAGGAGGAT-3’), *GNT-GBT0196-F1* (5’-GACTGAAGTCTGAGGACCTTGC-3’), *GNT-nrg2a-F1* (5’-gcagggaacagttaattgcttgacag-3’), *GNT-nrg2a-R4* (5’- ggctgaatcttggcaacaatgcaac -3’), *GNT-GBT0245-F1* (5’-CTTCTCAAATGGCTCCTTGCCTC-3’), *GNT-GBT0263-R1* (5’-TCCAACACCCTCCAACACC-3’), *GNT-GBT0261-F1* (5’-TATGGCTGTATATCATCACTGGTTGG-3’), *GNT-GBT0275-F2* (5’-TTAGAAACGGCAGGAAACCGAG-3’), *GNT-GBT0316-F1* (5’-TTGGTTACGTCCAGTGCGTC-3’), *GNT-SEC0325-F1* (5’-CCAGTCCGCCGCTGAGGG-3’), *GNT-SEC0325-R1* (5’-CGAATTGCTGTGAGATAGCGTTGAATAG-3’).

Transcripts from *nrg2a* alternate first exons, currently designated 1B and 1C, were manually identified and/or confirmed as follows. The presence of endogenous exon 1B transcript was confirmed using forward GS primer *RT-nrg2a-F3* (5’-ACACTTATCCATGCTGCTCATCGG-3’) with reverse GS primer in *nrg2a* exon 4 *RT-nrg2a-R3* (5’-CGTTGCATCTTCTGGCATGGC-3’) in 2 dpf wild-type cDNA. Exon 1C was identified via 5’RACE on wild-type RR-cDNA as previously described [17]. The presence of endogenous 1C transcript was confirmed using forward GS primer *RT-nrg2a-F3*.*1* (5’-AGGCGTAAGGACAGCCAAACTC-3’) with *RT-nrg2a-R3* in cDNA from *nrg2a* ^*+/+*^ and *nrg2a* ^*+/mn0237Gt*^ heterozygous (*nrg2a* ^*+/-*^) siblings. The presence of *nrg2a 1B*-*mRFP* and *1C*-*mRFP* fusion transcripts were confirmed using either GS primer *RT-nrg2a-F3* or *RT-nrg2a-F3*.*1*, respectively, with *mRFP* reverse primer *5R-mRFP-P2* in cDNA from *nrg2a* ^*+/mn0237Gt*^ heterozygous (*nrg2a* ^*+/-*^) and *nrg2a* ^*mn0237Gt/mn0237Gt*^ (*nrg2a* ^*-/-*^) homozygous mutant siblings. We were unable to confirm the presence of an endogenous transcript containing the predicted exon 1A. Embryos obtained from *nrg2a* ^*+/-*^ intercrosses were genotyped using three primers (*GNT-nrg2a-F1*, *GNT-nrg2a-R4*, and *5R-mRFP-P2*) in a single reaction, yielding either an 800 bp fragment for homozygous wild-types or a 1091 bp fragment for homozygous mutants, and both fragments for heterozygous siblings.

The isoform 1A sequence corresponds to predicted Nrg2a isoforms X7 –X9 (GenBank accession numbers XM_005161329, XM_005161330, XM_005161331) in Zv10 and to the sequence reported by Honjo *et al*. [51] (GenBank accession number NM_001099254). The isoform 1B sequence corresponds to those of predicted Nrg2a isoforms X1 –X4 (GenBank accession numbers NM_001099254, XM_009295459, XM_00929546, XM_005161327) in Zv10, and the isoform 1C sequence to that of predicted Nrg2a isoform X5 (GenBank accession number XM_009295461) in Zv10.

### Whole-mount *in situ* hybridization (WISH)

Larvae were fixed in 4% paraformaldehyde (PFA) and dehydrated/rehydrated through an ethanol/PBST series. Whole-mount *in situ* hybridization (WISH) was performed as previously described [57, 58, 118]. DIG-labeled probes were generated from cDNA clones as described in [44] for *fras1*, *hmcn1*, and *grip1*, in [61] for *msxc*, and in [17] for *mRFP*. cDNA clones for *ahrgef25b*, *fkbp10b*, and *erbb3a* were obtained from SourceBioScience, the cDNA clone for *egfra* was a gift from M. Sonawane. Probes for endogenous *nrg2a* were obtained with DIG-labeled *nrg2a* antisense riboprobe generated with primers *RT-nrg2a-F4* (5’-TCCAGTGTTGGCAGACGAAGG-3’) and *RT-nrg2a-R2* (5’-TTGGGCATGTATAACCGCAGGG-3’). For *nrg2a*, the *in situ* protocol was slightly modified from [118]. Nonspecific antibody binding was blocked with either blocking buffer (2% lamb serum (vol/vol), 2 mg/ml BSA) or 2% Boehringer Blocking Reagent (11096176001, Roche) in maleic acid buffer containing 0.1% Tween20 (MABT) for 4 h at room temperature prior to immunoreaction with anti-DIG-AP Fab fragments antibody (11093274910, Roche, 1:500). Fixed, stained WISH larvae were rehydrated through an ethanol/PBST series into 80% glycerol or into benzylbenzoate/benzylalcohol (2:1). Single images were obtained on a SteREO Lumar.V12 stereomicroscope (Zeiss) using AxioVision software (Zeiss). Single focal plane images for creating multi-image extended depth of field projections from brightfield images (“manual Z-stack”) were obtained using SCORE imaging techniques on an Axioplan2 microscope (Zeiss) with a PowerShot A640 camera (Canon) and ZoomBrowser EX software (Canon). Extended depth of field projections were generated in Image J (NIH) from multiple single focal plane images using StackReg and Extended Depth of Field plugins.

### Expression of Nrg2a-mRFP isoforms

To generate expression vectors pT3Ts-nrg2a-1B-mRFP and pT3Ts-nrg2a-1C-mRFP, the AUG-free mRFP coding sequence was amplified from pGBT-RP2 using primers *CDS-GBT_mRFP-F1* (5’- AGATCTCGCTAGCAATTATGGTTCAGCCGGAATTCACCC-3’) and *CDS-GBT_mRFP-R1* (5’-aaactagtcttaggctccggtggag-3’) and subcloned into a Strataclone vector backbone (Agilent). mRFP was then subcloned into the SpeI and BglII sites of pT3Ts-Cre [17] to create pT3Ts-mRFP. The *nrg2a*-*mRFP* isoforms were amplified from cDNA obtained from *nrg2a* ^*mn0237Gt/mn0237Gt*^ (*nrg2a* ^*-/-*^) homozygous mutant embryos using either forward primer *rob-nrg2a-1A* (5’-ATACTAGTATGCTGCTCATCGGGGT-3’) or *rob-nrg2a-1B* (5’-ATACTAGTATGTCAGAGGGTAAGAAGAAGGAAC-3’), respectively, with reverse primer *rob-nrg2a-R1* (5’-CCTCATCTTGATCTCGCCCTT-3’) and subcloned into the pT3Ts-mRFP backbone. The pT3TS-EGFPCAAX expression vector was created as follows: EGFPCAAX was amplified from 384.pME-EGFPCAAX plasmid [119] using primers *CDS_EGFP_CAAX-F1* (5’-GAAGGATCCATCATGGTGAGCAAGGG-3’) and *CDS-EGFP_CAAX-R1* (5’-CAAACTAGTCAGGAGAGCACACACTT-3’) and ligated into the SpeI and BglII site of pT3TS-Cre. To generate pT3TS_nlsTagBFP, a synthetic SV-40 nuclear localization sequence (NLS) was added to the N-terminus of Tag-BFP (pTagBFP-N, Evrogen, FP172), which was then cloned into the SpeI and BglII site of pT3TS-Cre. Expression vectors were linearized with SacI and RNA was made using the mMESSAGE mMACHINE kit (Ambion). 1 nL of 12.5 pg/nL *caax-EGFP*, 25 pg/nL *nls-BFP*, and 25 pg/nL of either *nrg2a-1B-mRFP* or *nrg2a-1C-mRFP* were co-injected into the yolks of one-cell stage wild-type embryos. 1 dpf injected embryos were imaged on a Zeiss LSM 780 confocal microscope.

### Sectioning of larvae

Larvae for cryosections were fixed in either 4% PFA or EAF (40% ethanol, 5% acetic acid, 4% formaldehyde in PBS) for 3h at RT, washed several times with PBST, embedded in 15% sucrose/1% agarose in PBS, incubated in 30% sucrose/PBS overnight and mounted in tissue freezing medium (Leica). 10 or 20 μm sections of tails were obtained using a Leica CM1850 cryostat. Larval heads were used for genotyping.

### Immuno- and histological staining

Immunostaining was performed essentially as previously described [120] using the following primary antibodies: anti-collagen II (DSHB II-II6B3-c, 1:200), rabbit anti-RFP (Abcam; ab62341, 1:200), chicken anti-GFP (Invitrogen; A10262, 1:300), mouse anti-p63 BC4A4 (Zytomend, 1:200), and mouse anti-MAP Kinase, activated (Sigma, M9692, 1:50). For pAkt staining, cryosections of EAF-fixed embryos were washed with PBS-TritonX, followed by an antigen retrieval in 10 mM Tris, 1 mM EDTA, pH 9.0 for 5 min at 98°C, blocking in 5% sheep serum and antibody incubation with rabbit anti-pAkt (S473) (Cell signaling #4060, 1:50) (modified after [121]). Secondary antibodies were anti-rabbitAlexa555, anti-mouseAlexa488, anti-mouseAlexa647, and anti-chickenAlexa488 (all Invitrogen, 1:200). Sections were mounted either directly in Mowiol containing DAPI or stained with a 1:1000 dilution of CellMask Deep Red Plasma membrane Stain (Molecular Probes) in PBS for 10 min and washed with PBS several times prior to mounting. Images were taken using a Zeiss Confocal (LSM710 META).

### EGFR/ErbB inhibitor treatment

A 10 mM stock of PD168393 (EMD Millipore Calbiochem #513033) was prepared with DMSO and diluted in embryo water to concentrations of 1 and 20 μM. 30 to 40 embryos were randomly selected for each treatment group and placed into Petri dishes (35–1007, BD Falcon). To examine the effect of inhibitor on the earliest stages of documented MFF phenotype through early pectoral fin fold development, PD168393 treatment and control groups were incubated from 24 hpf through 52 hpf. Larvae were gently transferred into fresh embryo water after incubation. Blinded treatment and control groups were and scored at 96 hpf with a Stemi 2000-C (Zeiss) dissecting microscope. Scoring was performed at 96 hpf to verify that inhibitor-induced morphological changes persisted beyond the end of the treatment window itself. Results were graphed with PRISM software (Graphpad); *p*-value and significance were calculated with Chi-square analysis. For TEM analysis samples, 20 μM-treated wild-type embryos were randomly selected immediately following incubation and removed into Trump’s fixative, as were age-matched sibling controls.

### Larval preparation for Transmission Electron Microscopy (TEM)

Embryos younger than 48 hpf were sorted by mRFP fluorescence. Larvae 48 hpf or older were sorted by MFF phenotype and fluorescence. Sorted or selected embryos were transferred into glass Petri dishes for all subsequent processing (Corning) and fixed in Trump’s fixative (4% formaldehyde, 1% glutaraldehyde in 0.1M phosphate buffer (pH 7.2)) for a minimum of 24 hr. Following fixation, zebrafish were secondarily fixed with 1% osmium tetroxide and 2% uranyl acetate, dehydrated through an ethanol series and embedded into Embed 812/Araldite resin. After a 24 hr polymerization at 60°C, ultrathin sections (0.1 μm) were stained with 2% uranyl acetate and lead citrate. Micrographs were acquired using a Jeol J1400 transmission electron microscope (JEOL, Inc., Peabody, MA) at 80 kV equipped with a Gatan digital CCD camera (Gatan, Inc., Warrendale, PA) and Digital Micrograph software. Minor adjustments were made in ImageJ or Keynote.

### Morpholino knockdown

Morpholino injections were performed according to standard protocols [11]. *lgl2* MO (5’-GCCCATGACGCCTGAACCTCTTCAT-3’) [54] (GeneTools); *p53* MO (5’-GCGCCATTGCTTTGCAAGAATTG-3’) (GeneTools). *p*-values were calculated with Chi-square analysis. Following phenotype evaluation, embryos were genotyped as described above.

## Supporting Information

S1 Fig mRFP fusion protein distribution in GBT lines recapitulates the endogenous expression patterns of *arhgef25b*, *fkbp10b*, and *msxc*. Panels (A, A’, C, C’, E, E’) show the same *in vivo* fluorescence images of mRFP localization in GBT lines *arhgef25b* ^mn0175Gt^ (A, A’), *fkbp10b* ^mn0316Gt^ (C, C’) and *msxc* ^mn0245Gt^ (E, E’) as in Fig 2E, 2F, 2K, 2L, 2M, and 2N, respectively. Panels underneath show images of wild-type embryos of comparable stages (indicated in hpf) and orientations following whole-mount *in situ* hybridization (WISH) with *arhgef25b* (B, B’), *fkbp10b* (D, D’), or *msxc* (F, F’) RNA probes. mRFP localization in the epidermis covering the yolk in the *arhgef25b* ^mn0175Gt^ gene-break allele (A) corresponds to endogenous *arhgef25b* gene expression (B). Both *arhgef25b* ^mn0175Gt^ mRFP localization and endogenous *arhgef25b* gene expression are also observed in the MFF (A’, B’). Furthermore, just as the gene-break alleles *fkbp10b* ^mn0316Gt^ (C, C’) and *msxc* ^mn0245Gt^ (E, E’) show mRFP localization in fin mesenchymal cells (FMCs), *fkbp10b* and *msxc* genes show endogenous FMC expression (C-F’; also compare with MsxC^mn0245Gt^-mRFP fusion protein localization in Fig 3E–3H). mRFP localization to the pectoral fin buds observed in the *fkbp10b* ^mn0316Gt^ allele (C) parallels endogenous *fkbp10b* expression in the pectoral fin buds (D). *fkbp10b* also shows expression in the posterior region of the notochord (D’). The weaker WISH signal in anterior (earlier specified) regions of the notochord compared to the mRFP signal (C’) points to transient expression of the endogenous gene in notochord cells, and higher stability of the GBT-generated mRFP fusion protein than the endogenous transcript. The endogenous *msxc* gene is also expressed in the maculae of the inner ear and in the pectoral fin buds (F), confirming that the *msxc* ^mn0245Gt^ GBT allele and its resulting mRFP fusion protein also recapitulate endogenous expression in tissues other than the skin (E).
(TIF)

S2 Fig Nucleotide and deduced amino acid sequences of the three N-terminal Nrg2a isoforms generated upon alternative exon 1 usage. (A) Nucleotide sequences of 5’ regions of three different *nrg2a* cDNA isoforms. Alternative exon 1A, 1B, and 1C sequences are in blue, shared exon 2 sequence is in black; start codon of isoform 1A is highlighted in light blue, start codons of isoforms 1B and 1C are highlighted in yellow. (B) Deduced amino acid sequences of N-termini of 1A, 1B, and 1C isoforms. Exon1B and exon1B-encoded sequences are in blue, exon2-encoded sequences in black, and the putative nuclear localization sequences (NLS) of the 1C isoform in red.
(TIF)

S3 Fig *nrg2a* mutant larvae have altered pectoral fin folds, lack swim bladders and die between 8 and 12 dpf. (A-D) Images of live larvae at 7 dpf. (A) Wild-type pectoral fin folds (PFF) typically follow a continuous arc such that the PFF edge lies close to the body when larvae are at rest. (B) PFFs in *nrg2a* mutant larvae (*mn0237Gt*/*mn0237Gt*) often depart from a continuous arc shape and/or have thickened edges similar to the MFF phenotype (arrowhead). In addition, wild-type larvae have well-developed swim bladders (C), but swim bladders fail to develop in *mn0237Gt*/*mn0237Gt* mutant larvae (D, arrowhead). (E) Graphical illustration of survival rates of *mn0237Gt*/*mn0237Gt* mutant larvae (n = 104 larvae per class).
(TIF)

S4 Fig Cleft cells in *nrg2a* mutants are largely unaffected, whereas ridge cells display expanded basal and reduced apical domains. Transmission electron micrographs (TEM) of distal-most region of dorsal MFF of wild-type (A) and *nrg2a* mutant (*mn0237Gt*/*mn0237Gt*) (B-D) embryos at 36 hpf (A, B) or 52 hpf (C, D). (A, B) Cleft cell (cc) morphogenesis creates the cleft, an invagination of the nascent dermal space (ds, red arrow) into the cleft cell. White lines trace cleft cell boundaries; red arrow termini (red arrowheads) indicate termination of the dermal space within the cleft. The *nrg2a* mutant (B) has an intact cleft cell with normal morphology. (C, D) Representative example of a ridge bulging into the dermal space, consisting of a single ridge cell with an extended basal border (blue; D) and a noticeably reduced apical border (red, D). Lateral borders are in white (D). For clarity, identical images are shown side by side with (D) and without (C) marked ridge cell borders. Magnification: 10,000X, scale bar: 2 μm. (A-D) 36 hpf; (E-F) 2 dpf. Abbreviatiations: cc, cleft cell; ds, dermal space; e, EVL cell; rc, ridge cell.
(TIF)

S5 Fig pERK levels in *nrg2a* mutant MFF basal keratinocytes are unchanged. Confocal images of whole-mount dorsal MFFs from *nrg2a* ^+/mn037Gt^ (A-D, I-K) and *nrg2a* ^mn037Gt/mn037Gt^ (E-H, M-P) embryos at 30hpf and 34 hpf do not reveal changes in activated ERK (phosphoERK, pERK) levels in ridge cells. Embryos were immunostained for phosphoErk (A, E, I, M) and basal keratinocytes were immunostained for Nrg2a-mRFP (B, F, J, N). Nuclei were counterstained with DAPI (C, G, K, O). Merged images are shown in D, H, K, and M. Arrows indicate ridge cells that are positive for pERK both in *nrg2a* ^+/mn037Gt^ and *nrg2a* ^mn037Gt/mn037Gt^ embryos.
(TIF)

## References

[1] Verhoeven EWM, Kraaimaat FW, van Weel C, van de Kerkhof PCM, Duller P, van der Valk PGM, et al Skin diseases in family medicine: prevalence and health care use. Annals of family medicine. 2008;6(4):349–54. 10.1370/afm.861 18626035 PMC2478509

[2] St Sauver JL, Warner DO, Yawn BP, Jacobson DJ, McGree ME, Pankratz JJ, et al Why patients visit their doctors: assessing the most prevalent conditions in a defined American population. Mayo Clinic proceedings Mayo Clinic. 2013;88(1):56–67. 10.1016/j.mayocp.2012.08.020 23274019 PMC3564521

[3] Bickers DR, Lim HW, Margolis D, Weinstock MA, Goodman C, Faulkner E, et al The burden of skin diseases: 2004. Journal of the American Academy of Dermatology. 2006;55(3):490–500. 10.1016/j.jaad.2006.05.048 16908356

[4] Finlay AY, Khan GK. Dermatology Life Quality Index (DLQI)—a simple practical measure for routine clinical use. Clinical and experimental dermatology. 1994;19(3):210–6. . 8033378 10.1111/j.1365-2230.1994.tb01167.x

[5] Chren MM, Lasek RJ, Quinn LM, Mostow EN, Zyzanski SJ. Skindex, a quality-of-life measure for patients with skin disease: reliability, validity, and responsiveness. The Journal of investigative dermatology. 1996;107(5):707–13. . 8875954 10.1111/1523-1747.ep12365600

[6] Finlay AY. The burden of skin disease: quality of life, economic aspects and social issues. Clinical medicine (London, England). 2009;9(6):592–4. . 20095308 10.7861/clinmedicine.9-6-592 PMC4952304

[7] Habif TP, Campbell J, James L, Chapman MS, Dinulos JGH, Zug KA. Skin Disease: Diagnosis and Treatment. 3rd ed: Elsevier Health Sciences; 2011 7 15 692 p.

[8] Li Q, Frank M, Thisse CI, Thisse BV, Uitto J. Zebrafish: a model system to study heritable skin diseases. Journal of Investigative Dermatology. 2011;131(3):565–71. 10.1038/jid.2010.388 21191402 PMC3342776

[9] Kettleborough RNW, Busch-Nentwich EM, Harvey SA, Dooley CM, de Bruijn E, van Eeden F, et al A systematic genome-wide analysis of zebrafish protein-coding gene function. Nature. 2013;496(7446):494–7. 10.1038/nature11992 23594742 PMC3743023

[10] Howe K, Clark MD, Torroja CF, Torrance J, Berthelot C, Muffato M, et al The zebrafish reference genome sequence and its relationship to the human genome. Nature. 2013;496(7446):498–503. 10.1038/nature12111 23594743 PMC3703927

[11] Nasevicius A, Ekker SC. Effective targeted gene ′knockdown′ in zebrafish. Nature Genetics. 2000;26(2):216–20. 10.1038/79951 . 11017081

[12] Keller PJ, Schmidt AD, Wittbrodt J, Stelzer EHK. Reconstruction of zebrafish early embryonic development by scanned light sheet microscopy. Science. 2008;322(5904):1065–9. 10.1126/science.1162493 . 18845710

[13] Haffter P, Granato M, Brand M, Mullins MC, Hammerschmidt M, Kane DA, et al The identification of genes with unique and essential functions in the development of the zebrafish, Danio rerio. Development. 1996;123:1–36. . 9007226 10.1242/dev.123.1.1

[14] Driever W, Solnica-Krezel L, Schier AF, Neuhauss SC, Malicki J, Stemple DL, et al A genetic screen for mutations affecting embryogenesis in zebrafish. Development. 1996;123:37–46. . 9007227 10.1242/dev.123.1.37

[15] Amsterdam A, Burgess S, Golling G, Chen W, Sun Z, Townsend K, et al A large-scale insertional mutagenesis screen in zebrafish. Genes & Development. 1999;13(20):2713–24. 10541557 10.1101/gad.13.20.2713 PMC317115

[16] Amsterdam A, Nissen RM, Sun Z, Swindell EC, Farrington S, Hopkins N. Identification of 315 genes essential for early zebrafish development. Proceedings of the National Academy of Sciences of the United States of America. 2004;101(35):12792–7. 10.1073/pnas.0403929101 15256591 PMC516474

[17] Clark KJ, Balciunas D, Pogoda H-M, Ding Y, Westcot SE, Bedell VM, et al In vivo protein trapping produces a functional expression codex of the vertebrate proteome. Nature methods. 2011;8(6):506–15. 10.1038/nmeth.1606 21552255 PMC3306164

[18] Ding Y, Sun X, Huang W, Hoage T, Redfield M, Kushwaha S, et al Haploinsufficiency of target of rapamycin attenuates cardiomyopathies in adult zebrafish. Circulation Research. 2011;109(6):658–69. 10.1161/CIRCRESAHA.111.248260 21757652 PMC3166359

[19] Ding Y, Liu W, Deng Y, Jomok B, Yang J, Huang W, et al Trapping cardiac recessive mutants via expression-based insertional mutagenesis screening. Circulation Research. 2013;112(4):606–17. 10.1161/CIRCRESAHA.112.300603 23283723 PMC3603352

[20] Liao H-K, Wang Y, Noack Watt KE, Wen Q, Breitbach J, Kemmet CK, et al Tol2 gene trap integrations in the zebrafish amyloid precursor protein genes appa and aplp2 reveal accumulation of secreted APP at the embryonic veins. Developmental Dynamics. 2012;241(2):415–25. 10.1002/dvdy.23725 22275008 PMC3448447

[21] Xu J, Gao J, Li J, Xue L, Clark KJ, Ekker SC, et al Functional analysis of slow myosin heavy chain 1 and myomesin-3 in sarcomere organization in zebrafish embryonic slow muscles. Journal of genetics and genomics = Yi chuan xue bao. 2012;39(2):69–80. 10.1016/j.jgg.2012.01.005 . 22361506 PMC3971575

[22] Petzold AM, Balciunas D, Sivasubbu S, Clark KJ, Bedell VM, Westcot SE, et al Nicotine response genetics in the zebrafish. Proceedings of the National Academy of Sciences of the United States of America. 2009;106(44):18662–7. 10.1073/pnas.0908247106 19858493 PMC2767365

[23] Kimmel CB, Warga RM, Schilling TF. Origin and organization of the zebrafish fate map. Development. 1990;108:581–581594. 2387237 10.1242/dev.108.4.581

[24] Fukazawa C, Santiago C, Park KM, Deery WJ, de la Torre Canny SG, Holterhoff CK, et al poky/chuk/ikk1 is required for differentiation of the zebrafish embryonic epidermis. Developmental Biology. 2010:1–12. 10.1016/j.ydbio.2010.07.037 20692251 PMC2956273

[25] Guzman A, Ramos-Balderas JL, Carrillo-Rosas S, Maldonado E. A stem cell proliferation burst forms new layers of P63 expressing suprabasal cells during zebrafish postembryonic epidermal development. Biology open. 2013;2(11):1179–86. 10.1242/bio.20136023 24244854 PMC3828764

[26] Lee RTH, Asharani PV, Carney TJ. Basal Keratinocytes Contribute to All Strata of the Adult Zebrafish Epidermis. PLoS ONE. 2014 10.1371/journal.pone.0084858.s005 . PMC3882266 24400120

[27] Fischer B, Metzger M, Richardson R, Knyphausen P, Ramezani T, Franzen R, et al p53 and TAp63 promote keratinocyte proliferation and differentiation in breeding tubercles of the zebrafish. PLoS Genetics. 2014;10(1):e1004048 10.1371/journal.pgen.1004048 24415949 PMC3886889

[28] Yang A, Kaghad M, Wang Y, Gillett E, Fleming MD, Dötsch V, et al p63, a p53 homolog at 3q27-29, encodes multiple products with transactivating, death-inducing, and dominant-negative activities. Molecular Cell. 1998;2(3):305–16. . 9774969 10.1016/s1097-2765(00)80275-0

[29] Yang A, Schweitzer R, Sun D, Kaghad M, Walker N, Bronson RT, et al p63 is essential for regenerative proliferation in limb, craniofacial and epithelial development. Nature. 1999;398(6729):714–8. 10.1038/19539 . 10227294

[30] Parsa R, Yang A, McKeon F, Green H. Association of p63 with proliferative potential in normal and neoplastic human keratinocytes. The Journal of investigative dermatology. 1999;113(6):1099–105. 10.1046/j.1523-1747.1999.00780.x . 10594758

[31] Mills AA, Zheng B, Wang XJ, Vogel H, Roop DR, Bradley A. p63 is a p53 homologue required for limb and epidermal morphogenesis. Nature. 1999;398(6729):708–13. 10.1038/19531 . 10227293

[32] Bakkers J, Hild M, Kramer C, Furutani-Seiki M, Hammerschmidt M. Zebrafish DeltaNp63 is a direct target of Bmp signaling and encodes a transcriptional repressor blocking neural specification in the ventral ectoderm. Developmental Cell. 2002;2(5):617–27. . 12015969 10.1016/s1534-5807(02)00163-6

[33] Lee H, Kimelman D. A dominant-negative form of p63 is required for epidermal proliferation in zebrafish. Developmental Cell. 2002;2(5):607–16. . 12015968 10.1016/s1534-5807(02)00166-1

[34] Romano R-A, Smalley K, Magraw C, Serna VA, Kurita T, Raghavan S, et al ΔNp63 knockout mice reveal its indispensable role as a master regulator of epithelial development and differentiation. Development. 2012;139(4):772–82. 10.1242/dev.071191 22274697 PMC3265062

[35] Coates MI. The origin of vertebrate limbs. Development. 1994;1994(Supplement):169–80. . 7579518

[36] Zhang X-G, Hou X-G. Evidence for a single median fin-fold and tail in the Lower Cambrian vertebrate, Haikouichthys ercaicunensis. Journal of evolutionary biology. 2004;17(5):1162–6. 10.1111/j.1420-9101.2004.00741.x . 15312089

[37] Dane PJ, Tucker JB. Modulation of epidermal cell shaping and extracellular matrix during caudal fin morphogenesis in the zebra fish Brachydanio rerio. Journal of embryology and experimental morphology. 1985;87:145–61. . 4031750

[38] Abe G, Ide H, Tamura K. Function of FGF signaling in the developmental process of the median fin fold in zebrafish. Developmental Biology. 2007;304(1):355–66. 10.1016/j.ydbio.2006.12.040 . 17258191

[39] Hadzhiev Y, Lele Z, Schindler S, Wilson SW, Ahlberg P, Strähle U, et al Hedgehog signaling patterns the outgrowth of unpaired skeletal appendages in zebrafish. BMC Developmental Biology. 2007;7:75 10.1186/1471-213X-7-75 17597528 PMC1950712

[40] Feitosa NM, Zhang J, Carney TJ, Metzger M, Korzh V, Bloch W, et al Hemicentin 2 and Fibulin 1 are required for epidermal-dermal junction formation and fin mesenchymal cell migration during zebrafish development. Developmental Biology. 2012;369(2):235–48. 10.1016/j.ydbio.2012.06.023 22771579 PMC3423513

[41] Wood A, Thorogood P. An analysis of in vivo cell migration during teleost fin morphogenesis. Journal of Cell Science. 1984;66:205–22. . 6746756 10.1242/jcs.66.1.205

[42] Durán I, Marí-Beffa M, Santamaría JA, Becerra J, Santos-Ruiz L. Actinotrichia collagens and their role in fin formation. Developmental Biology. 2011;354(1):160–72. 10.1016/j.ydbio.2011.03.014 . 21420398

[43] Webb AE, Sanderford J, Frank D, Talbot WS, Driever W, Kimelman D. Laminin α5 is essential for the formation of the zebrafish fins. Developmental Biology. 2007;311(2):369–82. 10.1016/j.ydbio.2007.08.034 . 17919534

[44] Carney TJ, Feitosa NM, Sonntag C, Slanchev K, Kluger J, Kiyozumi D, et al Genetic analysis of fin development in zebrafish identifies furin and hemicentin1 as potential novel fraser syndrome disease genes. PLoS Genetics. 2010;6(4):e1000907 10.1371/journal.pgen.1000907 20419147 PMC2855323

[45] Gautier P, Naranjo-Golborne C, Taylor MS, Jackson IJ, Smyth I. Expression of the fras1/frem gene family during zebrafish development and fin morphogenesis. Developmental Dynamics. 2008;237(11):3295–304. 10.1002/dvdy.21729 18816440

[46] Lee RTH, Knapik EW, Thiery JP, Carney TJ. An exclusively mesodermal origin of fin mesenchyme demonstrates that zebrafish trunk neural crest does not generate ectomesenchyme. Development. 2013;140(14):2923–32. 10.1242/dev.093534 23739134 PMC3699280

[47] McGregor L, Makela V, Darling SM, Vrontou S, Chalepakis G, Roberts C, et al Fraser syndrome and mouse blebbed phenotype caused by mutations in FRAS1/Fras1 encoding a putative extracellular matrix protein. Nature Genetics. 2003;34(2):203–8. 10.1038/ng1142 . 12766769

[48] Vrontou S, Petrou P, Meyer BI, Galanopoulos VK, Imai K, Yanagi M, et al Fras1 deficiency results in cryptophthalmos, renal agenesis and blebbed phenotype in mice. Nature Genetics. 2003;34(2):209–14. 10.1038/ng1168 . 12766770

[49] Jadeja S, Smyth I, Pitera JE, Taylor MS, van Haelst M, Bentley E, et al Identification of a new gene mutated in Fraser syndrome and mouse myelencephalic blebs. Nature Genetics. 2005;37(5):520–5. 10.1038/ng1549 15838507

[50] Postel R, Margadant C, Fischer B, Kreft M, Janssen H, Secades P, et al Kindlin-1 mutant zebrafish as an in vivo model system to study adhesion mechanisms in the epidermis. Journal of Investigative Dermatology. 2013;133(9):2180–90. 10.1038/jid.2013.154 . 23549420

[51] Honjo Y, Kniss J, Eisen JS. Neuregulin-mediated ErbB3 signaling is required for formation of zebrafish dorsal root ganglion neurons. Development. 2008;135(15):2615–25. 10.1242/dev.022178 18599505 PMC2756296

[52] Reischauer S, Levesque MP, Nusslein-Volhard C, Sonawane M. Lgl2 executes its function as a tumor suppressor by regulating ErbB signaling in the zebrafish epidermis. PLoS Genetics. 2009;5(11):e1000720 10.1371/journal.pgen.1000720 19911055 PMC2771016

[53] Olayioye MA, Neve RM, Lane HA, Hynes NE. The ErbB signaling network: receptor heterodimerization in development and cancer. Embo J. 2000;19(13):3159–67. . 10880430 10.1093/emboj/19.13.3159 PMC313958

[54] Sonawane M, Carpio Y, Geisler R, Schwarz H, Maischein H-M, Nuesslein-Volhard C. Zebrafish penner/lethal giant larvae 2 functions in hemidesmosome formation, maintenance of cellular morphology and growth regulation in the developing basal epidermis. Development. 2005;132(14):3255–65. 10.1242/dev.01904 . 15983403

[55] Clark KJ, Argue DP, Petzold AM, Ekker SC. zfishbook: connecting you to a world of zebrafish revertible mutants. Nucleic acids research. 2012;40(Database issue):D907–11. 10.1093/nar/gkr957 22067444 PMC3245101

[56] van Eeden FJ, Granato M, Schach U, Brand M, Furutani-Seiki M, Haffter P, et al Genetic analysis of fin formation in the zebrafish, Danio rerio. Development. 1996;123:255–62. . 9007245 10.1242/dev.123.1.255

[57] Thisse C, Thisse B. High-resolution in situ hybridization to whole-mount zebrafish embryos. Nature Protocols. 2008;3(1):59–69. 10.1038/nprot.2007.514 . 18193022

[58] Bedell VM, Person AD, Larson JD, McLoon A, Balciunas D, Clark KJ, et al The lineage-specific gene ponzr1 is essential for zebrafish pronephric and pharyngeal arch development. Development. 2012;139(4):793–804. 10.1242/dev.071720 22274699 PMC3265064

[59] Metcalfe WK, Kimmel CB, Schabtach E. Anatomy of the posterior lateral line system in young larvae of the zebrafish. The Journal of Comparative Neurology. 1985;233(3):377–89. 10.1002/cne.902330307 . 3980776

[60] Choo BGH, Kondrichin I, Parinov S, Emelyanov A, Go W, Toh W-c, et al Zebrafish transgenic Enhancer TRAP line database (ZETRAP). BMC Developmental Biology. 2006;6:5 10.1186/1471-213X-6-5 16478534 PMC1386650

[61] Ekker M, Akimenko MA, Bremiller R, Westerfield M. Regional expression of three homeobox transcripts in the inner ear of zebrafish embryos. Neuron. 1992;9(1):27–35. . 1352984 10.1016/0896-6273(92)90217-2

[62] Feitosa NM, Richardson R, Bloch W, Hammerschmidt M. Basement membrane diseases in zebrafish. Methods in cell biology. 2011;105:191–222. 10.1016/B978-0-12-381320-6.00008-4 . 21951531

[63] Takamiya K, Kostourou V, Adams S, Jadeja S, Chalepakis G, Scambler PJ, et al A direct functional link between the multi-PDZ domain protein GRIP1 and the Fraser syndrome protein Fras1. Nature Genetics. 2004;36(2):172–7. 10.1038/ng1292 . 14730302

[64] Vogel MJ, van Zon P, Brueton L, Gijzen M, van Tuil MC, Cox P, et al Mutations in GRIP1 cause Fraser syndrome. Journal of Medical Genetics. 2012;49(5):303–6. 10.1136/jmedgenet-2011-100590 . 22510445

[65] Schanze D, Kayserili H, Satkın BN, Altunoglu U, Zenker M. Fraser syndrome due to mutations in GRIP1—clinical phenotype in two families and expansion of the mutation spectrum. American Journal of Medical Genetics Part A. 2014;164A(3):837–40. 10.1002/ajmg.a.36343 . 24357607

[66] Vogel BE, Hedgecock EM. Hemicentin, a conserved extracellular member of the immunoglobulin superfamily, organizes epithelial and other cell attachments into oriented line-shaped junctions. Development. 2001;128(6):883–94. . 11222143 10.1242/dev.128.6.883

[67] Abreu-Velez AM, Howard MS. Collagen IV in Normal Skin and in Pathological Processes. North American journal of medical sciences. 2012;4(1):1–8. 10.4103/1947-2714.92892 22393540 PMC3289483

[68] Hashimoto T, Amagai M, Parry DA, Dixon TW, Tsukita S, Tsukita S, et al Desmoyokin, a 680 kDa keratinocyte plasma membrane-associated protein, is homologous to the protein encoded by human gene AHNAK. Journal of Cell Science. 1993;105 (Pt 2):275–86. . 8408266 10.1242/jcs.105.2.275

[69] Masunaga T, Shimizu H, Ishiko A, Fujiwara T, Hashimoto T, Nishikawa T. Desmoyokin/AHNAK protein localizes to the non-desmosomal keratinocyte cell surface of human epidermis. The Journal of investigative dermatology. 1995;104(6):941–5. . 7769263 10.1111/1523-1747.ep12606213

[70] Dear TN, Meier NT, Hunn M, Boehm T. Gene structure, chromosomal localization, and expression pattern of Capn12, a new member of the calpain large subunit gene family. Genomics. 2000;68(2):152–60. 10.1006/geno.2000.6289 . 10964513

[71] Nassar D, Letavernier E, Baud L, Aractingi S, Khosrotehrani K. Calpain activity is essential in skin wound healing and contributes to scar formation. PLoS ONE. 2012;7(5):e37084 10.1371/journal.pone.0037084 22615899 PMC3353912

[72] Radoja N, Gazel A, Banno T, Yano S, Blumenberg M. Transcriptional profiling of epidermal differentiation. Physiological genomics. 2006;27(1):65–78. 10.1152/physiolgenomics.00031.2006 . 16822832

[73] Carney TJ, von der Hardt S, Sonntag C, Amsterdam A, Topczewski J, Hopkins N, et al Inactivation of serine protease Matriptase1a by its inhibitor Hai1 is required for epithelial integrity of the zebrafish epidermis. Development. 2007;134(19):3461–71. 10.1242/dev.004556 . 17728346

[74] Webb AE, Driever W, Kimelman D. psoriasis regulates epidermal development in zebrafish. Developmental Dynamics. 2008;237(4):1153–64. 10.1002/dvdy.21509 . 18351656

[75] Slanchev K, Carney TJ, Stemmler MP, Koschorz B, Amsterdam A, Schwarz H, et al The epithelial cell adhesion molecule EpCAM is required for epithelial morphogenesis and integrity during zebrafish epiboly and skin development. PLoS Genetics. 2009;5(7):e1000563 10.1371/journal.pgen.1000563.s010 . 19609345 PMC2700972

[76] Dodd ME, Hatzold J, Mathias JR, Walters KB, Bennin DA, Rhodes J, et al The ENTH domain protein Clint1 is required for epidermal homeostasis in zebrafish. Development. 2009;136(15):2591–600. 10.1242/dev.038448 19570844 PMC2709065

[77] Petersen TN, Brunak S, von Heijne G, Nielsen H. SignalP 4.0: discriminating signal peptides from transmembrane regions. Nat Methods. 2011;8(10):785–6. 10.1038/nmeth.1701 21959131

[78] Brameier M, Wiuf C. Ab initio identification of human microRNAs based on structure motifs. BMC Bioinformatics. 2007;8:478 Epub 2007/12/20. 1471-2105-8-478 [pii] 10.1186/1471-2105-8-478 18088431 PMC2238772

[79] Gong Z, Ju B, Wang X, He J, Wan H, Sudha PM, et al Green fluorescent protein expression in germ-line transmitted transgenic zebrafish under a stratified epithelial promoter from keratin8. Dev Dyn. 2002;223(2):204–15. 11836785 10.1002/dvdy.10051

[80] Carraway KL, Weber JL, Unger MJ, Ledesma J, Yu N, Gassmann M, et al Neuregulin-2, a new ligand of ErbB3/ErbB4-receptor tyrosine kinases. Nature. 1997;387(6632):512–6. 10.1038/387512a0 . 9168115

[81] Chang H, Riese DJ, Gilbert W, Stern DF, McMahan UJ. Ligands for ErbB-family receptors encoded by a neuregulin-like gene. Nature. 1997;387(6632):509–12. 10.1038/387509a0 . 9168114

[82] Hobbs SS, Coffing SL, Le ATD, Cameron EM, Williams EE, Andrew M, et al Neuregulin isoforms exhibit distinct patterns of ErbB family receptor activation. Oncogene. 2002;21(55):8442–52. 10.1038/sj.onc.1205960 . 12466964

[83] Rojas-Muñoz A, Rajadhyksha S, Gilmour D, van Bebber F, Antos C, Rodríguez Esteban C, et al ErbB2 and ErbB3 regulate amputation-induced proliferation and migration during vertebrate regeneration. Developmental Biology. 2009;327(1):177–90. 10.1016/j.ydbio.2008.12.012 . 19133254

[84] Citri A, Skaria KB, Yarden Y. The deaf and the dumb: the biology of ErbB-2 and ErbB-3. Exp Cell Res. 2003;284(1):54–65. . 12648465 10.1016/s0014-4827(02)00101-5

[85] Fedi P, Pierce JH, di Fiore PP, Kraus MH. Efficient coupling with phosphatidylinositol 3-kinase, but not phospholipase C gamma or GTPase-activating protein, distinguishes ErbB-3 signaling from that of other ErbB/EGFR family members. Mol Cell Biol. 1994;14(1):492–500. . 8264617 10.1128/mcb.14.1.492-500.1994 PMC358399

[86] Wiley HS. Trafficking of the ErbB receptors and its influence on signaling. Exp Cell Res. 2003;284(1):78–88. . 12648467 10.1016/s0014-4827(03)00002-8

[87] Cook RS, Garrett JT, Sanchez V, Stanford JC, Young C, Chakrabarty A, et al ErbB3 ablation impairs PI3K/Akt-dependent mammary tumorigenesis. Cancer Res. 2011;71(11):3941–51. 10.1158/0008-5472.CAN-10-3775 21482676 PMC3204389

[88] Wu X, Chen Y, Li G, Xia L, Gu R, Wen X, et al Her3 is associated with poor survival of gastric adenocarcinoma: Her3 promotes proliferation, survival and migration of human gastric cancer mediated by PI3K/AKT signaling pathway. Med Oncol. 2014;31(4):903 10.1007/s12032-014-0903-x 24623015

[89] Mechler BM, McGinnis W, Gehring WJ. Molecular cloning of lethal(2)giant larvae, a recessive oncogene of Drosophila melanogaster. The EMBO Journal. 1985;4(6):1551–7. 3928370 10.1002/j.1460-2075.1985.tb03816.x PMC554381

[90] Tanentzapf G, Tepass U. Interactions between the crumbs, lethal giant larvae and bazooka pathways in epithelial polarization. Nature Cell Biology. 2003;5(1):46–52. 10.1038/ncb896 . 12510193

[91] Amagai M. A Mystery of AHNAK/Desmoyokin Still Goes On. Journal of Investigative Dermatology. 2004;123(4):xiv–xiv. 10.1111/j.0022-202X.2004.23432.x . 15373799

[92] Longo I, Scala E, Mari F, Caselli R, Pescucci C, Mencarelli MA, et al Autosomal recessive Alport syndrome: an in-depth clinical and molecular analysis of five families. Nephrology, dialysis, transplantation: official publication of the European Dialysis and Transplant Association—European Renal Association. 2006;21(3):665–71. 10.1093/ndt/gfi312 . 16338941

[93] Talbot JC, Walker MB, Carney TJ, Huycke TR, Yan Y-L, BreMiller RA, et al fras1 shapes endodermal pouch 1 and stabilizes zebrafish pharyngeal skeletal development. Development. 2012;139(15):2804–13. 10.1242/dev.074906 22782724 PMC3392706

[94] Mohideen M-APK, Beckwith LG, Tsao-Wu GS, Moore JL, Wong ACC, Chinoy MR, et al Histology-based screen for zebrafish mutants with abnormal cell differentiation. Developmental Dynamics. 2003;228(3):414–23. 10.1002/dvdy.10407 . 14579380

[95] Sabaliauskas NA, Foutz CA, Mest JR, Budgeon LR, Sidor AT, Gershenson JA, et al High-throughput zebrafish histology. Methods. 2006;39(3):246–54. . 16870470 10.1016/j.ymeth.2006.03.001

[96] Falls DL. Neuregulins: functions, forms, and signaling strategies. Exp Cell Res. 2003;284(1):14–30. . 12648463 10.1016/s0014-4827(02)00102-7

[97] Walter P, Lingappa VR. Mechanism of protein translocation across the endoplasmic reticulum membrane. Annu Rev Cell Biol. 1986;2:499–516. . 3030381 10.1146/annurev.cb.02.110186.002435

[98] Friedlander M, Blobel G. Bovine opsin has more than one signal sequence. Nature. 1985;318(6044):338–43. . 2999609 10.1038/318338a0

[99] Stachowiak MK, Moffett J, Maher P, Tucholski J, Stachowiak EK. Growth factor regulation of cell growth and proliferation in the nervous system. A new intracrine nuclear mechanism. Mol Neurobiol. 1997;15(3):257–83. . 9457702 10.1007/BF02740663

[100] Forthmann B, Grothe C, Claus P. A nuclear odyssey: fibroblast growth factor-2 (FGF-2) as a regulator of nuclear homeostasis in the nervous system. Cell Mol Life Sci. 2015 . 25552245 10.1007/s00018-014-1818-6 PMC11113852

[101] Marti U, Ruchti C, Kampf J, Thomas GA, Williams ED, Peter HJ, et al Nuclear localization of epidermal growth factor and epidermal growth factor receptors in human thyroid tissues. Thyroid. 2001;11(2):137–45. . 11288982 10.1089/105072501300042785

[102] Bryant DM, Stow JL. Nuclear translocation of cell-surface receptors: lessons from fibroblast growth factor. Traffic. 2005;6(10):947–54. . 16138907 10.1111/j.1600-0854.2005.00332.x

[103] Lin SY, Makino K, Xia W, Matin A, Wen Y, Kwong KY, et al Nuclear localization of EGF receptor and its potential new role as a transcription factor. Nat Cell Biol. 2001;3(9):802–8. . 11533659 10.1038/ncb0901-802

[104] Britto JM, Lukehurst S, Weller R, Fraser C, Qiu Y, Hertzog P, et al Generation and characterization of neuregulin-2-deficient mice. Mol Cell Biol. 2004;24(18):8221–6. . 15340081 10.1128/MCB.24.18.8221-8226.2004 PMC515040

[105] Slamon DJ, Godolphin W, Jones LA, Holt JA, Wong SG, Keith DE, et al Studies of the HER-2/neu proto-oncogene in human breast and ovarian cancer. Science. 1989;244(4905):707–12. . 2470152 10.1126/science.2470152

[106] Hardy KM, Booth BW, Hendrix MJ, Salomon DS, Strizzi L. ErbB/EGF signaling and EMT in mammary development and breast cancer. J Mammary Gland Biol Neoplasia. 2010;15(2):191–9. 10.1007/s10911-010-9172-2 20369376 PMC2889136

[107] Larue L, Bellacosa A. Epithelial-mesenchymal transition in development and cancer: role of phosphatidylinositol 3' kinase/AKT pathways. Oncogene. 2005;24(50):7443–54. . 16288291 10.1038/sj.onc.1209091

[108] Zimmermann T, Kashyap A, Hartmann U, Otto G, Galle PR, Strand S, et al Cloning and characterization of the promoter of Hugl-2, the human homologue of Drosophila lethal giant larvae (lgl) polarity gene. Biochemical and Biophysical Research Communications. 2008;366(4):1067–73. 10.1016/j.bbrc.2007.12.084 . 18155665

[109] Lyons DA, Pogoda H-M, Voas MG, Woods IG, Diamond B, Nix R, et al erbb3 and erbb2 are essential for schwann cell migration and myelination in zebrafish. Current biology: CB. 2005;15(6):513–24. 10.1016/j.cub.2005.02.030 . 15797019

[110] Budi EH, Patterson LB, Parichy DM. Embryonic requirements for ErbB signaling in neural crest development and adult pigment pattern formation. Development. 2008;135(15):2603–14. 10.1242/dev.019299 18508863 PMC2704560

[111] Wansbury O, Panchal H, James M, Parry S, Ashworth A, Howard B. Dynamic expression of Erbb pathway members during early mammary gland morphogenesis. Journal of Investigative Dermatology. 2008;128(4):1009–21. 10.1038/sj.jid.5701118 . 17960183

[112] Dunn M, Sinha P, Campbell R, Blackburn E, Levinson N, Rampaul R, et al Co-expression of neuregulins 1, 2, 3 and 4 in human breast cancer. The Journal of Pathology. 2004;203(2):672–80. 10.1002/path.1561 . 15141382

[113] Marshall C, Blackburn E, Clark M, Humphreys S, Gullick WJ. Neuregulins 1–4 are expressed in the cytoplasm or nuclei of ductal carcinoma (in situ) of the human breast. Breast Cancer Research and Treatment. 2006;96(2):163–8. 10.1007/s10549-005-9073-z . 16261396

[114] Cooper MS, Szeto DP, Sommers-Herivel G, Topczewski J, Solnica-Krezel L, Kang H-C, et al Visualizing morphogenesis in transgenic zebrafish embryos using BODIPY TR methyl ester dye as a vital counterstain for GFP. Developmental Dynamics. 2005;232(2):359–68. 10.1002/dvdy.20252 . 15614774

[115] Petzold AM, Bedell VM, Boczek NJ, Essner JJ, Balciunas D, Clark KJ, et al SCORE imaging: specimen in a corrected optical rotational enclosure. Zebrafish. 2010;7(2):149–54. 10.1089/zeb.2010.0660 . 20528262 PMC3117241

[116] Liu YG, Whittier RF. Thermal asymmetric interlaced PCR: automatable amplification and sequencing of insert end fragments from P1 and YAC clones for chromosome walking. Genomics. 1995;25(3):674–81. . 7759102 10.1016/0888-7543(95)80010-j

[117] Parinov S, Kondrichin I, Korzh V, Emelyanov A. Tol2 transposon-mediated enhancer trap to identify developmentally regulated zebrafish genes in vivo. Developmental Dynamics. 2004;231(2):449–59. 10.1002/dvdy.20157 . 15366023

[118] Jacobs NL, Albertson RC, Wiles JR. Using whole mount in situ hybridization to link molecular and organismal biology. J Vis Exp. 2011;(49). . 21490578 10.3791/2533 PMC3197306

[119] Kwan KM, Fujimoto E, Grabher C, Mangum BD, Hardy ME, Campbell DS, et al The Tol2kit: a multisite gateway-based construction kit for Tol2 transposon transgenesis constructs. Dev Dyn. 2007;236(11):3088–99. Epub 2007/10/17. 10.1002/dvdy.21343 . 17937395

[120] Hammerschmidt M, Pelegri F, Mullins MC, Kane DA, van Eeden FJ, Granato M, et al dino and mercedes, two genes regulating dorsal development in the zebrafish embryo. Development. 1996;123:95–102. . 9007232 10.1242/dev.123.1.95

[121] van der Velden YU, Wang L, Zevenhoven J, van Rooijen E, van Lohuizen M, Giles RH, et al The serine-threonine kinase LKB1 is essential for survival under energetic stress in zebrafish. Proc Natl Acad Sci U S A. 2011;108(11):4358–63. 10.1073/pnas.1010210108 21368212 PMC3060253

[122] Gonzalez-Mariscal L, Tapia R, Chamorro D. Crosstalk of tight junction components with signaling pathways. Biochim Biophys Acta. 2008;1778(3):729–56. . 17950242 10.1016/j.bbamem.2007.08.018

