# Supplementary figures and images for: Protein-Trap Insertional Mutagenesis Uncovers New Genes Involved in Zebrafish Skin Development, Including a Neuregulin 2a-Based ErbB Signaling Pathway Required during Median Fin Fold Morphogenesis
Source: PLoS One. 2015 Jun 25;10(6):e0130688. doi: 10.1371/journal.pone.0130688 (PMC4482254; doi:10.1371/journal.pone.0130688)

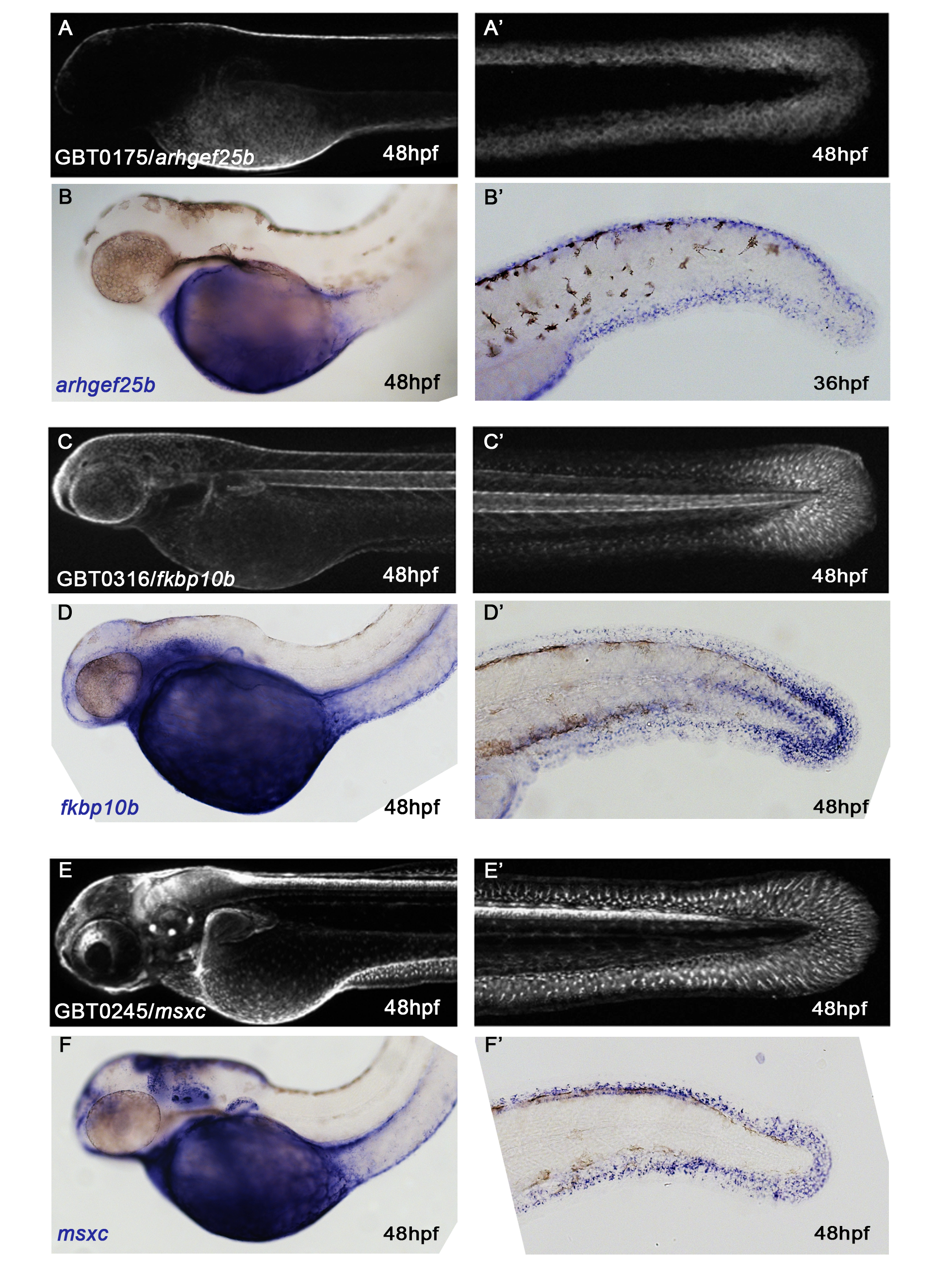

Supplement: S1 Fig — Panels (A, A’, C, C’, E, E’) show the same in vivo fluorescence images of mRFP localization in GBT lines arhgef25b mn0175Gt (A, A’), fkbp10b mn0316Gt (C, C’) and msxc mn0245Gt (E, E’) as in Fig 2E, 2F, 2K, 2L, 2M, and 2N, respectively. Panels underneath show images of wild-type embryos of comparable stages (indicated in hpf) and orientations following whole-mount in situ hybridization (WISH) with arhgef25b (B, B’), fkbp10b (D, D’), or msxc (F, F’) RNA probes. mRFP localization in the epidermis covering the yolk in the arhgef25b mn0175Gt gene-break allele (A) corresponds to endogenous arhgef25b gene expression (B). Both arhgef25b mn0175Gt mRFP localization and endogenous arhgef25b gene expression are also observed in the MFF (A’, B’). Furthermore, just as the gene-break alleles fkbp10b mn0316Gt (C, C’) and msxc mn0245Gt (E, E’) show mRFP localization in fin mesenchymal cells (FMCs), fkbp10b and msxc genes show endogenous FMC expression (C-F’; also compare with MsxCmn0245Gt-mRFP fusion protein localization in Fig 3E–3H). mRFP localization to the pectoral fin buds observed in the fkbp10b mn0316Gt allele (C) parallels endogenous fkbp10b expression in the pectoral fin buds (D). fkbp10b also shows expression in the posterior region of the notochord (D’). The weaker WISH signal in anterior (earlier specified) regions of the notochord compared to the mRFP signal (C’) points to transient expression of the endogenous gene in notochord cells, and higher stability of the GBT-generated mRFP fusion protein than the endogenous transcript. The endogenous msxc gene is also expressed in the maculae of the inner ear and in the pectoral fin buds (F), confirming that the msxc mn0245Gt GBT allele and its resulting mRFP fusion protein also recapitulate endogenous expression in tissues other than the skin (E). (TIF) [file pone.0130688.s001.tif]

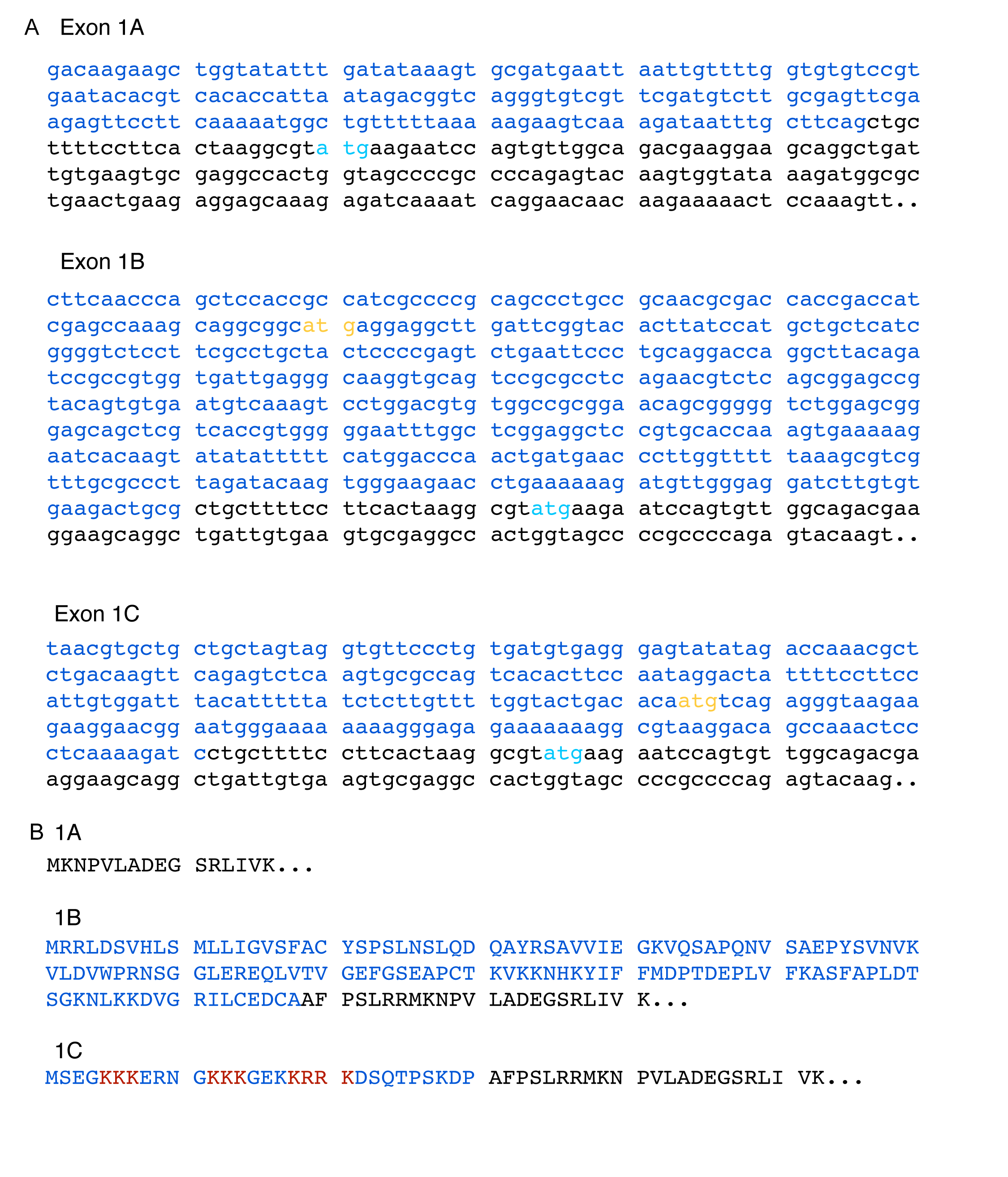

Supplement: S2 Fig — (A) Nucleotide sequences of 5’ regions of three different nrg2a cDNA isoforms. Alternative exon 1A, 1B, and 1C sequences are in blue, shared exon 2 sequence is in black; start codon of isoform 1A is highlighted in light blue, start codons of isoforms 1B and 1C are highlighted in yellow. (B) Deduced amino acid sequences of N-termini of 1A, 1B, and 1C isoforms. Exon1B and exon1B-encoded sequences are in blue, exon2-encoded sequences in black, and the putative nuclear localization sequences (NLS) of the 1C isoform in red. (TIF) [file pone.0130688.s002.tif]

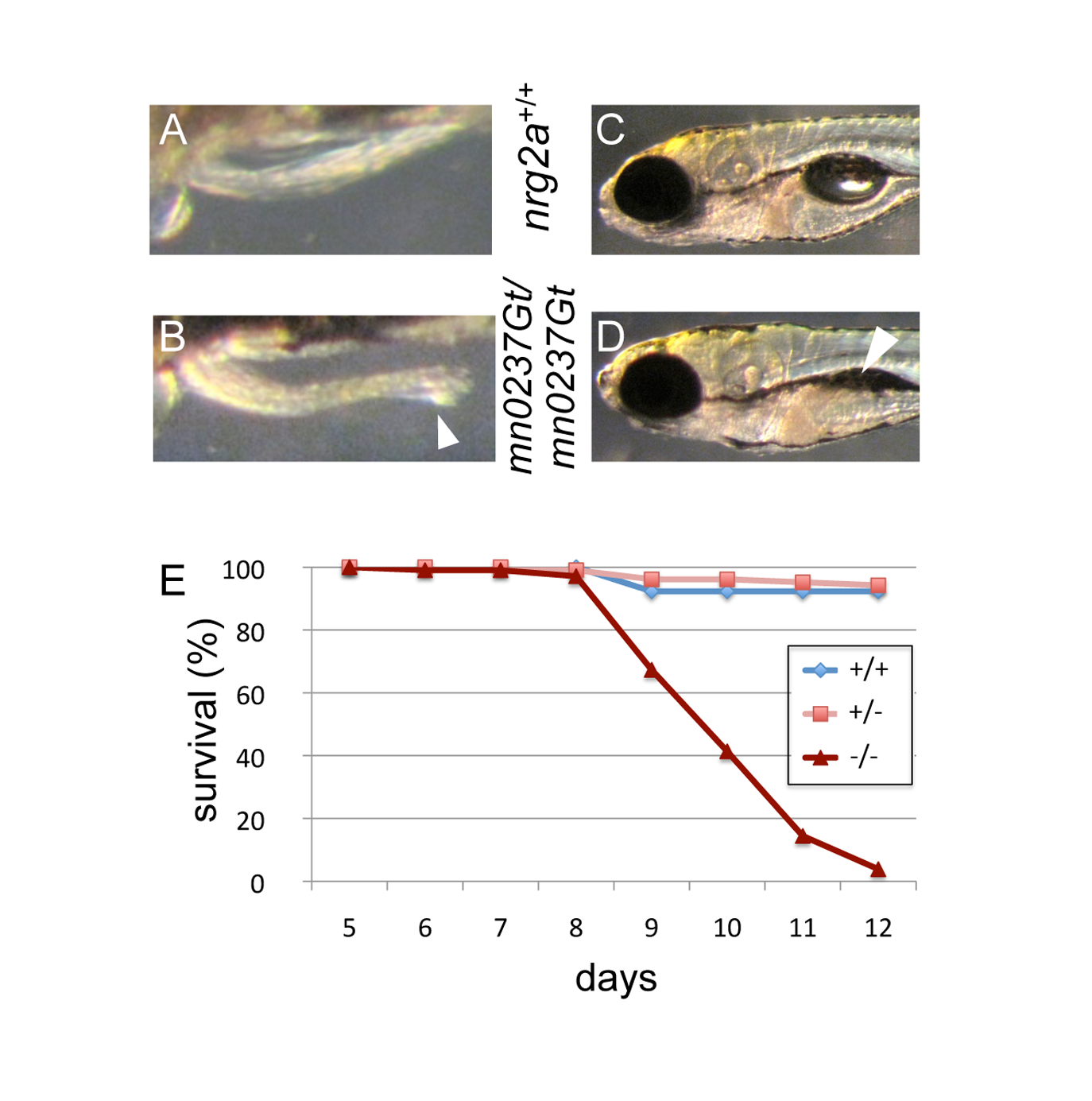

Supplement: S3 Fig — (A-D) Images of live larvae at 7 dpf. (A) Wild-type pectoral fin folds (PFF) typically follow a continuous arc such that the PFF edge lies close to the body when larvae are at rest. (B) PFFs in nrg2a mutant larvae (mn0237Gt/mn0237Gt) often depart from a continuous arc shape and/or have thickened edges similar to the MFF phenotype (arrowhead). In addition, wild-type larvae have well-developed swim bladders (C), but swim bladders fail to develop in mn0237Gt/mn0237Gt mutant larvae (D, arrowhead). (E) Graphical illustration of survival rates of mn0237Gt/mn0237Gt mutant larvae (n = 104 larvae per class). (TIF) [file pone.0130688.s003.tif]

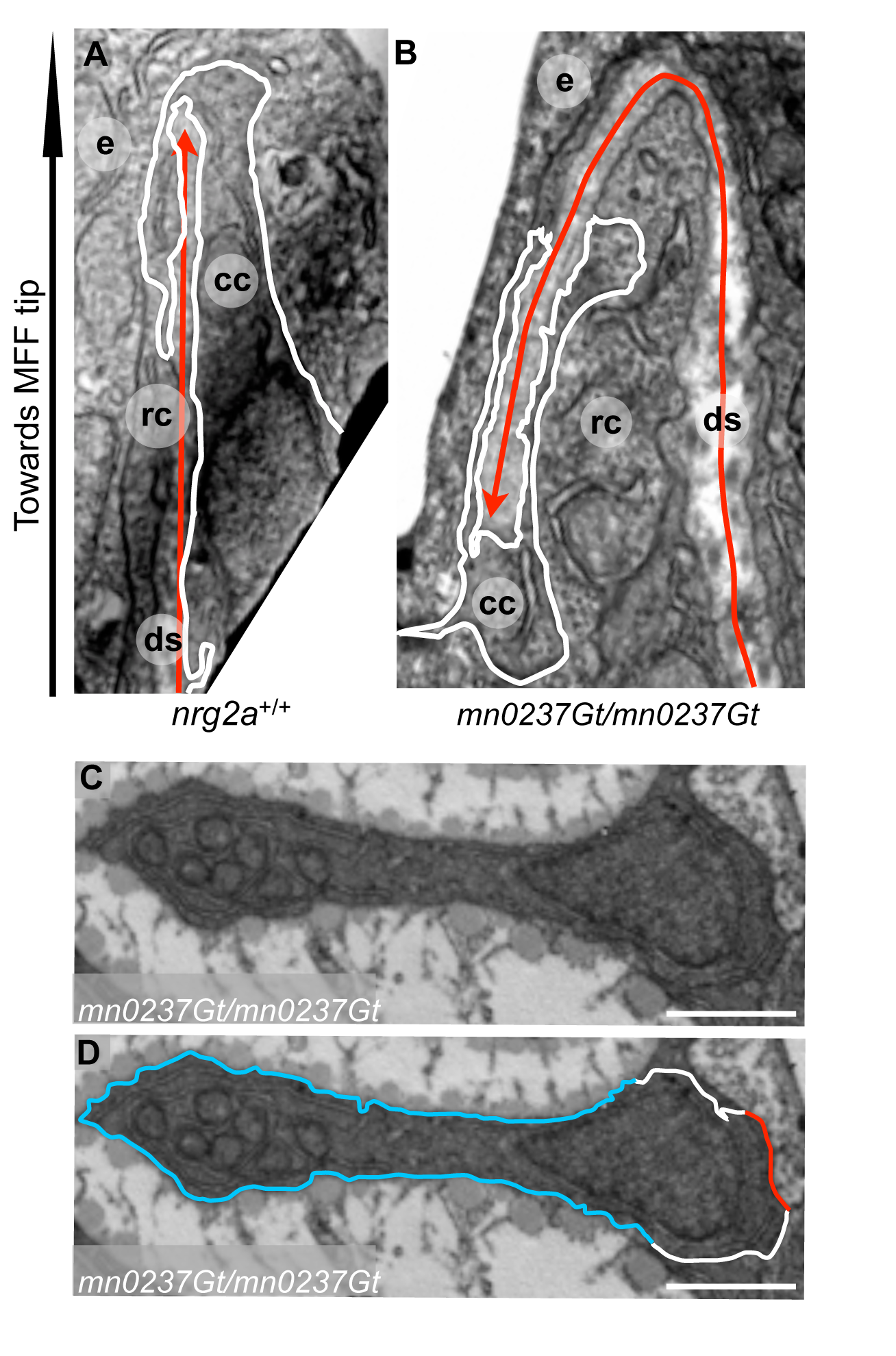

Supplement: S4 Fig — Transmission electron micrographs (TEM) of distal-most region of dorsal MFF of wild-type (A) and nrg2a mutant (mn0237Gt/mn0237Gt) (B-D) embryos at 36 hpf (A, B) or 52 hpf (C, D). (A, B) Cleft cell (cc) morphogenesis creates the cleft, an invagination of the nascent dermal space (ds, red arrow) into the cleft cell. White lines trace cleft cell boundaries; red arrow termini (red arrowheads) indicate termination of the dermal space within the cleft. The nrg2a mutant (B) has an intact cleft cell with normal morphology. (C, D) Representative example of a ridge bulging into the dermal space, consisting of a single ridge cell with an extended basal border (blue; D) and a noticeably reduced apical border (red, D). Lateral borders are in white (D). For clarity, identical images are shown side by side with (D) and without (C) marked ridge cell borders. Magnification: 10,000X, scale bar: 2 μm. (A-D) 36 hpf; (E-F) 2 dpf. Abbreviatiations: cc, cleft cell; ds, dermal space; e, EVL cell; rc, ridge cell. (TIF) [file pone.0130688.s004.tif]

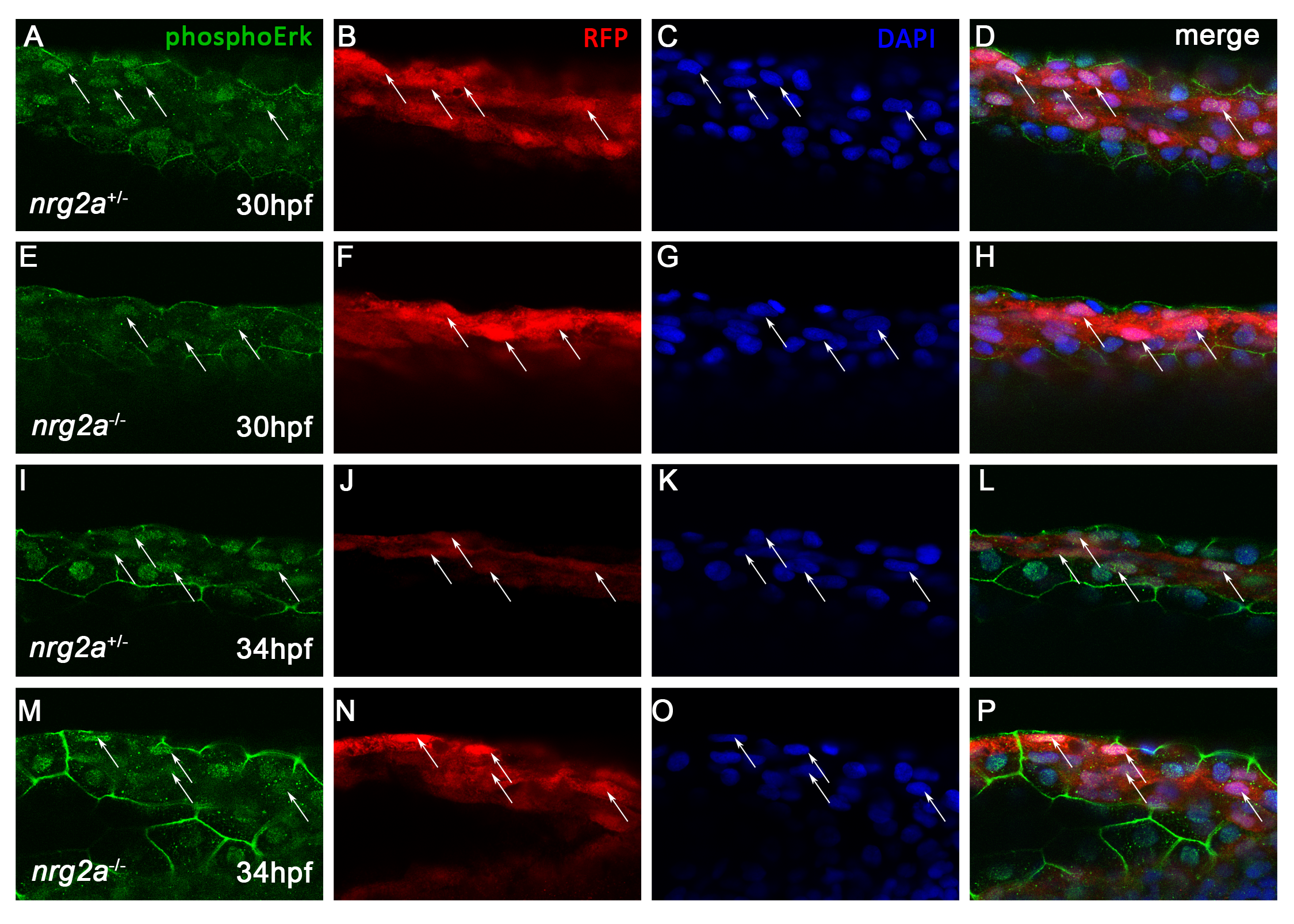

Supplement: S5 Fig — Confocal images of whole-mount dorsal MFFs from nrg2a +/mn037Gt (A-D, I-K) and nrg2a mn037Gt/mn037Gt (E-H, M-P) embryos at 30hpf and 34 hpf do not reveal changes in activated ERK (phosphoERK, pERK) levels in ridge cells. Embryos were immunostained for phosphoErk (A, E, I, M) and basal keratinocytes were immunostained for Nrg2a-mRFP (B, F, J, N). Nuclei were counterstained with DAPI (C, G, K, O). Merged images are shown in D, H, K, and M. Arrows indicate ridge cells that are positive for pERK both in nrg2a +/mn037Gt and nrg2a mn037Gt/mn037Gt embryos. (TIF) [file pone.0130688.s005.tif]
